# Function Analysis of *MBF1*, a Factor Involved in the Response to Amino Acid Starvation and Virulence in *Candida albicans*

**DOI:** 10.3389/ffunb.2021.658899

**Published:** 2021-03-15

**Authors:** Sara Amorim-Vaz, Alix T. Coste, Van Du T. Tran, Marco Pagni, Dominique Sanglard

**Affiliations:** ^1^Institute of Microbiology, Lausanne University Hospital, Lausanne, Switzerland; ^2^Vital-IT Group, SIB Swiss Institute of Bioinformatics, Lausanne, Switzerland

**Keywords:** *Candida albicans*, virulence, Mbf1, Gcn4, starvation

## Abstract

*Candida albicans* is a commensal of human mucosae, but also one of the most common fungal pathogens of humans. Systemic infections caused by this fungus, mostly affecting immunocompromised patients, are associated to fatality rates as high as 50% despite the available treatments. In order to improve this situation, it is necessary to fully understand how *C. albicans* is able to cause disease and how it copes with the host defenses. Our previous studies have revealed the importance of the *C. albicans* gene *MBF1* in virulence and ability to colonize internal organs of mammalian and insect hosts. *MBF1* encodes a putative transcriptional regulator, and as such it likely has an impact in the regulation of *C. albicans* gene expression during host infection. Here, recent advances in RNA-seq technologies were used to obtain a detailed analysis of the impact of *MBF1* on *C. albicans* gene expression both *in vitro* and during infection. *MBF1* was involved in the regulation of several genes with a role in glycolysis and response to stress, particularly to nutritional stress. We also investigated whether an interaction existed between *MBF1* and *GCN4*, a master regulator of response to starvation, and found that both genes were needed for resistance to amino acid starvation, suggesting some level of interaction between the two. Reinforcing this idea, we showed that the proteins encoded by both genes could interact. Consistent with the role of *MBF1* in virulence, we also established that *GCN4* was necessary for virulence in the mouse model of systemic infection as well as in the *Galleria mellonella* infection model.

## Introduction

*Candida albicans* is a fungal commensal of mucosae in the majority of humans, but it is also an opportunistic pathogen. It can cause mucosal infections or even invade bloodstream and internal organs if conditions are favorable. Examples of such conditions are immunosuppression of the host due to disease or medical treatments, or imbalance of the microbial flora associated for instance to the use of broad-spectrum antibiotics (Odds, 1988). Mucosal infections by *C. albicans* such as oral thrush or vulvovaginal candidiasis, although not life-threatening, are associated to high morbidity. Systemic infections by *C. albicans*, on the other hand, have mortality rates that reach 50%, highlighting the inefficacy of available antifungal treatments (Falagas et al., 2006; Leroy et al., 2009; Pfaller and Diekema, 2010). For the improvement of treatments, it is essential to better understand *C. albicans* biology and its interaction with the host.

One of the most remarkable characteristics of *C. albicans* is its ability to propagate in such diverse environments as the oral mucosa, the gastrointestinal tract, internal organs like kidneys or the brain, or the bloodstream. This implies a great capacity for adapting cellular metabolism to changing environmental conditions, including different nutrient availability and interaction with different components of the immune system. Adaptation of metabolism depends on the fine-tuning of gene expression, which in turn depends on the correct functioning of transcriptional regulators. In a previous study we identified a new gene, *MBF1*, which is necessary for full virulence of *C. albicans* in both a mouse and an invertebrate model of systemic candidiasis (Amorim-Vaz et al., 2015a). *MBF1* is predicted to encode a transcriptional coactivator. Besides this prediction, based on homology to proteins in other organisms, not much information is available on *MBF1* and its precise role in *C. albicans* virulence is not yet understood. In the same above-mentioned study (Amorim-Vaz et al., 2015a), a battery of *in vitro* tests was carried out, including several types of stresses thought to be relevant during infection, with the goal of understanding which pathways are regulated by this gene. Intriguingly, no clear phenotype was found in the *in vitro* conditions tested.

*MBF1* is conserved among eukaryotes (Takemaru et al., 1997; Dragoni et al., 1998; Smith et al., 1998; Aravind and Koonin, 1999; Zegzouti et al., 1999). It was first identified in the silkworm *Bombyx mori* bridging a transcription factor, BmFTZ-F1, to the TATA-binding protein (TBP) (Li et al., 1994), hence its classification as a transcriptional coactivator. Since then, it has been investigated in several other organisms and shown to bridge different transcription factors, containing sequence-specific DNA-binding domains, to TBP, but not to bind directly to DNA (reviewed in de Koning et al., 2009), suggesting it functions by recruiting TBP to promoters where transcription factors are found (Ozaki et al., 1999). Structural analyses were able to precise that the C-terminal end corresponds to a well-structured helix-turn-helix (HTH) motif (Mishima et al., 1999; Ozaki et al., 1999).

Regarding its function, *MBF1* has been associated to stress response in different organisms. In yeasts, it has been studied in *Saccharomyces cerevisiae* and shown to participate in the response to nutritional stress, i.e., amino acid starvation, by bridging physically TBP to the transcription factor Gcn4 (Takemaru et al., 1998). In addition, *MBF1* was also suggested to be involved in translation fidelity by suppressing frameshift mutations in *S. cerevisiae*. Recent studies have revealed that Mbf1 is associated with ribosomal complexes and other proteins (Rps3 and Acs1) to inhibit frameshifts when mRNAs are translated (Wang et al., 2018). In the pathogenic yeast *Cryptococcus neoformans MBF1* was necessary for activation of the laccase gene that mediates melanization and virulence (Walton et al., 2005). In the filamentous fungus *Beauveria bassiana*, a pathogen of insects, *MBF1* was also shown to bind TBP and its deletion affected hyphal morphogenesis, stress tolerance and virulence (Ying et al., 2014; Song et al., 2015).

Here, we investigated the function of *MBF1* in *C. albicans* and attempted to understand its contribution to virulence. Transcriptional analyses *in vitro* and *in vivo* suggest that *MBF1* is implicated in resistance to stress in *C. albicans* and in particular to nutritional stress. *In vitro* phenotypic tests revealed the importance of this gene in resistance to amino acid starvation. We investigated a possible interaction of Mbf1 with Gcn4, a master regulator of the response to starvation in *C. albicans* and showed physical interaction between both proteins. Contrary to previous published works (Brand et al., 2004), we showed that *GCN4* was necessary for virulence in a mouse model of systemic infection as well as in an insect model, highlighting the importance of resistance to nutritional stresses during infection.

## Materials and Methods

### Primers and Plasmids

The primers and plasmids used in this study are listed in Supplementary Tables 1, 2 (Data Sheet 6.docx).

### Strains and Media

*Candida albicans* strains used in this study are listed in Supplementary Table 3 (Data Sheet 6.docx) and included the wild type laboratory strain SC5314 as well as mutants derived from this strain that were constructed during this project, including ACY367 used in Amorim-Vaz et al. (2015a). Strains were grown in the following media (when grown on solid media, 2% agar (Difco) was added): complete medium yeast extract peptone dextrose (YEPD): 1% Bacto peptone (Difco Laboratories, Basel, Switzerland), 0.5% Yeast extract (Difco) and 2% glucose (Fluka, Buchs, Switzerland); YEPD supplemented with 200 μg/ml nourseothricin (Werner BioAgents); minimal medium yeast nitrogen base (YNB): YNB (Difco) and 2% glucose (Fluka); YNB supplemented with complete supplement mixture (CSM) (MP Biomedicals); YNB supplemented with CSM without histidine (-his) (MP Biomedicals) with or without the addition of 3-Amino-1,2,4-triazole (3-AT) (Sigma) (1 to 5 mM, specified later in the text), YNB supplemented with histidine and leucine (YNB +his +leu) or with histidine only (YNB +his) and supplemented with cysteine and methionine (each 2.5 mM).

*Escherichia coli* DH5α was used as a host for plasmid constructions and propagation. DH5α was grown in LB broth or LB-agar plates, supplemented with ampicillin (0.1 mg/ml) or chloramphenicol (0.1 mg/ml) when required.

### Construction of Mutant Strains

For construction of the marker-free full ORF deletion mutant of *GCN4*, the *C. albicans* SC5314 strain was used as the parental strain. The 5′- and 3′-untranslated regions (UTR) of the gene were amplified by PCR using a *Taq* DNA polymerase (New England Biolabs) and primers GCN4-knp, GCN4-Xho, GCN4-SacII, and GCN4-SacI (Supplementary Table 1). The resulting fragments were introduced in pSFS2A (Reuss et al., 2004), which contains the *SAT1*-flipping cassette including *SAT1* (conferring resistance to nourseothricin) controlled by the *ACT1* promoter, as well as an FLP-recombinase gene controlled by a maltose-inducible promoter, all flanked by *FRT* sequences. The resulting deletion plasmid, pDS1911 (Supplementary Table 2), was digested by KpnI and SacI and used to delete the first allele of *GCN4* using the yeast transformation protocol described in Sanglard et al. (1996). Transformants were allowed to grow on YEPD-agar containing nourseothricin (200 μg/ml) for 24–48 h at 35°C. Isolated transformants were incubated in liquid YEP medium containing maltose (2%) for at least 4 h under agitation at 30°C. Nourseothricin-susceptible clones were selected on agar plates of YEPD medium containing only 15 μg/ml nourseothricin for 24 h at 35°C. The second allele was next deleted using the same deletion cassette, followed by the same process to obtain nourseothricin-susceptible clones. The clones obtained were controlled by PCR to confirm the absence of wild type allele using a Taq DNA polymerase (New England Biolabs) and primers GCN4-kpn and GCN4-SacI.

The *MBF1* mutant used here was described in Amorim-Vaz et al. (2015a). A new revertant strain was constructed for this mutant, as well as for the *gcn4*Δ/Δ mutant. For construction of revertant strains for *MBF1* and *GCN4*, the wild type alleles were amplified by PCR from SC5314. For *MBF1*, primers orf19.3294-5F-Apa and orf19.3294-3R-Xho (Supplementary Table 1) were used to contain 450-bp and 420-bp 5′- and 3′-UTRs, respectively. The resulting fragment was introduced in the *MBF1* deletion cassette (pAC296) by ApaI-XhoI digestion, resulting in pSV5. For *GCN4* reversion, primers Gcn4-kpn and Gcn4-XhoIrev2 (Supplementary Table 1) were used to contain 490-bp and 440-bp 5′- and 3′-UTRs, respectively. The resulting fragment was introduced in the *GCN4* deletion cassette (pDS1911) by KpnI-XhoI digestion, resulting in pDS1917. The reversion cassettes were sequenced using primers 3294-60 for *MBF1* and GCN_seq_+350F and GCN4_seq_+800R for GCN4 (Supplementary Table 1) to verify the sequence integrity of the genes of interest. Plasmid pSV5, which contains the *MBF1* wild type allele, was digested by ApaI and SacI, and plasmid pDS1917, which contains the *GCN4* wild type allele, was digested by KpnI and BssHII. The resulting fragments were transformed according to the method described in Sanglard et al. (1996) into the corresponding mutant strains (ACY367 for *MBF1*, DSY4843 for *GCN4*), replacing one of the mutated alleles by homologous recombination. Transformants were again selected on YEPD-nourseothricin (200 μg/ml). To create the double mutant for *MBF1* and *GCN4* genes, the same procedure described above for construction of the *GCN4* deletion mutant was applied on the *MBF1* mutant strain (ACY367). The resulting strain (DSY4917) was then transformed sequentially with the reversion cassettes of both *MBF1* and *GCN4*, as described above, to obtain a double revertant. All mutant or revertant strains were verified by Southern blot [Supplementary Figure 1 (Data Sheet 7.docx)].

The *MBF1*-*GFP* fusion strain constructed here contained a green fluorescent protein (GFP) fused to Mbf1 at the C-terminal end. The *MBF1* ORF was amplified from SC5314 with primers MBF1_atg_SalI and MBF1_nostop_sph (Supplementary Table 1) and was cloned into compatible SalI-SphI sites of pDS1202 (Coste et al., 2004), a plasmid containing *GFP* and *URA3* as a selective marker, to yield pSV17. The final Mbf1-Gfp fusion was under the control of the *ACT1* promoter. Plasmid pSV17 was digested by StuI and transformed into SVY25, a strain lacking both *MBF1* and *URA3*, yielding SVY26.

### Serial Dilution Assay

Yeast cultures were grown overnight in liquid YEPD, diluted 1/100 into YNB –His, grown for 3 h and finally diluted to a concentration of 2.5 × 10^7^ cells/ml. Four 10-fold serial dilutions in PBS (phosphate buffered saline: 137 mM NaCl, 2.7 mM KCl, 4.3 mM Na_2_HPO_4_, 1.47 mM KH_2_PO_4_) were prepared at a final concentration of 2.5 × 10^2^ cells/ml. Four microliters of each dilution were spotted onto agar plates of YNB + CSM, YNB -His or YNB -His+3-AT (5 mM) and incubated for 24–72 h at 35°C.

### Growth Curves

Overnight *C. albicans* cultures were washed twice with PBS, and diluted in 100 μL of the indicated media for an initial optical density (OD) value at 540 nm (OD_540_) of 0.1 in a 96-well plate (Corning^®^ Half-area 96 Well Plate, polystyrene). The plate was kept at 30°C without agitation. OD_540_ was measured every 10 min for 24 h, with 3 s of shaking before each measure in a FLUOstar Omega microplate reader (BMG Labtech). Growth curves and doubling times were calculated using GraphPad Prism (7.00).

### Visualization of GFP Fluorescent Cells

Strains for visualization of GFP-MBF1 were grown on different media including YNB + CSM, YNB -CSM or YNB –CSM + 3-AT 1 mM. Cells were washed twice in PBS, resuspended in 1 ml PBS and stained with Hoechst 33342 (6.8 μg/ml) for 20 min in the dark at room temperature. Then, cells were again washed twice in PBS and resuspended in PBS, and 3 μl of cell suspension were mixed with 3 μl of Mowiol. Fluorescence (GFP and Hoechst) was revealed by microscopy. Fluorescence microscopy and phase-contrast microscopy were performed with a Zeiss Axioplan microscope equipped for epifluorescence microscopy with a 100-W mercury high-pressure bulb. Images obtained with a SPOT RT3 cooled 2 Mp charge-coupled-device (CCD) camera (Diagnostic Instruments, Inc., MI, USA) were recorded and captured with VisiView (Visitron Systems GmbH, Germany).

### Bimolecular Fluorescence Complementation

In order to address the potential interaction of *MBF1* with *GCN4*, a bimolecular fluorescence complementation tool was used based on the reconstitution of yeast-enhanced monomeric Venus (yEmVenus) according to Subotic et al. (2017). *MBF1* and *GCN4* ORFs were amplified with primers pairs MBF1_BiFC_NF/MBF1_BiFC_NR and BiFC-GCN4-NF/BiFC-GCN4-NR (Supplementary Table 1) to be introduced in BiFC1 and BiFC3 digested by *Xma*I and *Asc*I following the In-Fusion protocol (Takara Bio, Saint-Germain-en-Laye, France). The resulting plasmids were named pDS2121 (BiFC3-derived containing N-terminal tagging of *MBF1* with the C-terminal end of yEmVenus, CV-MBF1) and pDS2126 (BiFC1-derived containing N-terminal tagging of *GCN4* with the N-terminal end of yEmVenus, NV-MBF1). pDS2121 and parent plasmid BiFC3 were digested by *Not*I and first introduced in strain SN152 (Noble and Johnson, 2005) as described (Subotic et al., 2017) and transformants selected in YNB +his +leu medium supplemented by methionine and cysteine in order to repress the *MET3-*dependent expression of fusion proteins. The resulting strains were DSY5529 (CV-MBF1) and DSY5552 (CV). pDS2126 and BiFC1 were digested by *Not*I and introduced in strain DSY5529 and transformants selected in YNB +his supplemented by methionine and cysteine to result in strain DSY5544 (CV-MBF1, NV-GCN4) and DSY5554 (CV-MBF1, NV). BiFC1 was introduced the same way in strain DSY5552 to obtain DSY5551 (CV, NV-GCN4). To induce the protein fusions repressed by the *MET3* promoter, cultures were first pre-grown overnight in YEPD medium. Hundred microliter of the pre-cultures were washed with YNB and inoculated in YNB -his for 3–4 h at 30°C under constant shaking. Cells were next visualized for reconstituted yEmVenus with a Zeiss Axioplan microscope but equipped with a filter set 46 HE for yellow fluorescence and images were recorded as above-mentioned.

### Mouse Infection Experiments and Ethics Statement

All animal experiments were performed at the University of Lausanne and at the University Hospital Centre under the surveillance and with the approval of the institutional Animal Use Committee: Affaires Vétérinaires du Canton de Vaud, Switzerland; with the authorization n° 1734.3, according to the decree 18 of the federal law on animal protection. For all mice experiments, female BALB/c mice (6 weeks old; Charles River France) were housed in ventilated cages with free access to food and water. Strains used for infections were grown in individual tubes overnight under agitation at 30°C in YEPD. Each strain was then diluted 100-fold in YEPD and grown again overnight under agitation at 30°C. Overnight cultures were washed twice in PBS and resuspended in 5 ml PBS. The concentration of each culture was measured by optical density at 540 nm and each strain was diluted in PBS to the desired concentrations.

For fungal burden quantification experiments, groups of seven mice were used. Mice were injected through the lateral tail vein with 250 μl of a cell suspension containing 8 x 10^5^ cells/ml, corresponding to 2 × 10^5^ cells per animal. Three days post-infection, kidneys were recovered and processed for CFU counting as described previously (Vandeputte et al., 2011). Statistical analyses of CFU differences were performed using the Mann-Whitney test. For mice survival experiments, groups of nine to 10 mice were used. Mice were injected through the lateral tail vein with 250 μl of a cell suspension containing 2 × 10^6^ cells/ml. Animals were monitored daily for their temperature, weight and health state. The post-infection day of natural death or of euthanasia was recorded for each animal. Survival experiments ended 14 to 21 days after infection. Statistical analyses of survival data were done using the log-rank (Mantel-Cox) test.

### *Galleria mellonella* Infection Experiments

*Galleria mellonella* larvae were purchased from Bait Express GmbH (Basel, Switzerland). Upon arrival, the larvae were stored at 15°C in the dark with wood shavings and used within a maximum of 1 week. Larvae with a weight ranging from 350 to 400 mg were used for the experiments. For fungal burden quantification experiments, groups of 10 to 12 larvae were used. Each larva was injected through the last left proleg with 40 μl of a cell suspension containing 5 × 10^6^ cells/ml using a Myjector U-100 insulin syringe (Terumo Europe) as described in Amorim-Vaz et al. (2015a). A control group was included which was injected with 40 μl of sterile PBS, as well as a group of non-injected larvae. Larvae were kept at 30°C in the dark and sacrificed 24 h post-infection and processed for CFU counting as described in Amorim-Vaz et al. (2015a). Strains were tested twice in *G. mellonella*, thus representing a minimum of 20 infected larvae for each strain. Statistical analyses of CFU differences were performed using the Mann-Whitney test. For survival experiments, groups of 15 to 24 larvae were used. Larvae were inoculated with 40 μl of a *C. albicans* cell suspension containing 1.25 × 10^7^ cells/ml and treated as described in Amorim-Vaz et al. (2015a). Survival experiments ended 14 to 21 days after infection and were repeated twice. Statistical analyses of survival data were carried out using the log-rank (Mantel-Cox) test.

### Infection of Mice for RNA Extraction

For infection of mice with the purpose of extracting RNA from infected kidneys, *C. albicans* strains were grown overnight under agitation at 30°C in YEPD, then diluted 100-fold in YEPD and grown until the approximate density of 1.5 × 10^7^ cells/ml (measured by optical density at 540 nm). Cultures were then washed twice in PBS and resuspended in 5 ml PBS. The concentration of the culture was measured by optical density and diluted in PBS to the concentration of 2 × 10^6^ cells/ml. Mice were injected through the tail vein with 250 μl of cell suspension and sacrificed 16 and 48 h post-infection. Kidneys were collected, halved longitudinally and immediately placed in vials containing 1 ml of RNAlater solution (Life Technologies) to immediately stabilize RNA. Samples were kept on ice and then at −80°C until the time of RNA extraction.

### RNA Extractions

When preparing cell suspensions for infection of mice or larvae, 50 ml of the 1.5 x 10^7^ cell/ml suspensions were kept for direct RNA extraction of the *in vitro* culture. *Candida albicans in vitro* cultured cells were washed twice in PBS and then flash-frozen with liquid nitrogen (N_2_).

After mice sacrifice, mice kidneys were transferred from the RNAlater solution to a 10 cm diameter mortar and flash-frozen with N_2_. All samples were then processed as described in Amorim-Vaz et al. (2015a).

For RNA extractions from *in vitro* cultures of SC5314 (wild type), ACY367 (*mbf1*Δ/Δ) and DSY4843 (*gcn4*Δ/Δ) in the presence or absence of amino acid starvation, cells were grown overnight in 5 ml YEPD and washed twice with PBS. After resuspension of cells in PBS, optical density was measured and the appropriate dilutions were made in order to obtain 2 × 10^6^ cells/mL, either in 50 mL of YNB supplemented with CSM, in 50 mL of YNB -his or in 50 mL of YNB -his with 3-AT. Triplicates were included for each strain in each condition. Cells were then grown at 30°C with agitation for ~3.5 h, then centrifuged at 30°C for 5 min at 5,000 g and the cell pellet immediately resuspended in 500 μL of RNAlater^®^. These cells were kept at −80°C until the time of RNA extraction.

Frozen samples from infected and non-infected tissues were thawed on ice, centrifuged at low speed to remove the RNAlater^®^ and the cells resuspended in 300 μL of Trizol^®^ (Life Technologies), 300 μl of phenol-chloroform-isoamyl alcohol 25-24-1 (Sigma), 300 μl of RNA buffer (0.1 M Tris HCl pH 7.5, 0.1 M LiCl, 10 mM EDTA, 0.5% SDS) and the equivalent to 200 μl of acid-washed 1 mm-diameter glass beads in a screw-capped RNase-free 2 ml tube. Hereafter the samples were treated in the same way as the *in vivo* RNA samples (see above).

### RNA-Seq Library Preparation, Sequencing, and Data Processing

For RNA samples extracted from mouse kidneys and the corresponding *in vitro* samples, RNA libraries for RNA-seq were prepared using the SureSelect^*XT*^ RNA Target Enrichment for Illumina Multiplexed Sequencing kit (Agilent Technologies) as described in Amorim-Vaz et al. (2015b). The set of oligonucleotide baits used for capture of *C. albicans* transcripts, as well as the RNA-sequencing and data analysis processes were also described in Amorim-Vaz et al. (2015b). RNA-sequencing was performed by the Lausanne Genomics Technologies Facility (LGTF) in an Illumina HiSeq 2500 system. Data analysis up to differential gene expression was performed by the Vital-IT Group at the Swiss Institute of Bioinformatics (SIB) as described in Amorim-Vaz et al. (2015b). Briefly, sequencing data were processed using Illumina Pipeline software version 1.84. Purity-filtered reads were adapters- and quality trimmed with Cutadapt (Martin, 2011) and filtered for low complexity with Prinseq (Schmieder and Edwards, 2011). Reads were aligned against the *C. albicans* genome SC5314 version A21-s02-m09-r07 using TopHat2 (Kim et al., 2013). The number of read counts per gene locus was summarized with htseqcount (Anders et al., 2015). The read count data were normalized with the TMM (trimmed mean of M-values) method available in the R Bioconductor package edgeR (Robinson and Oshlack, 2010) and subsequently transformed to log2 counts per million by voom, a method implemented in the R bioconductor package limma (Law et al., 2014). The RNA-seq libraries used to analyze the transcriptomes of *MBF1* and *GCN4* mutants during amino acid starvation were prepared by the LGTF. RNA-seq libraries were prepared using 500 ng of total RNA and the Illumina TruSeq Stranded mRNA Library Prep Kit reagents (Catalog Number RS-122-2101; Illumina, San Diego, California, USA) on a Sciclone liquid handling robot (PerkinElmer; Waltham, Massachusetts, USA) using a PerkinElmer-developed automated script. Cluster generation was performed with the resulting libraries using the Illumina TruSeq SR Cluster Kit v4 reagents (Catalog Number GD-401-4001) and sequenced on the Illumina HiSeq 2500 using TruSeq SBS Kit V4 reagents (Catalog Number FC-401-4002). Sequencing data were processed using the Illumina Pipeline Software version 1.84 and further analyzed as described above. All transcriptomic data can be found under Bioproject PRJNA350368.

### Validation of RNA Sequencing Data by qPCR

To validate RNA-seq data, expression levels of 6 genes (*BIO2, HWP1, RBT1, STP4, YWP1*, and *ZRT1*) were determined by qPCR as described in Amorim-Vaz et al. (2015b) using the primers listed in Supplementary Table 1. The expression level of *ACT1* was used for normalization. All reactions were performed in duplicate and repeated twice.

### Statistics

Statistics used in this work (log-rank tests, Mann-Whitney tests, ROUT analysis) were carried out using Graph Prism (software 8.4.3) unless specifically described in the text. Gene set enrichment analysis (GSEA) were performed with a GSEA software (4.1.0). GSEA analysis parameters were as follows: norm, meandiv; scoring_scheme, weighted; set_min, 15; nperm, 1000; set_max, 500. GSEA results were uploaded into Cytoscape 3.0 with the following parameters: *P*-value cut-off, 0.01; FDR q-value, 0.05.

## Results

### *MBF1*-Dependent Gene Expression *in vitro*

*In silico* predictions performed on *MBF1* conclude that the C-terminal domain exhibits DNA-binding properties (Kelley et al., 2015). Accordingly, *MBF1* is annotated in the Candida Genome Database as a transcriptional coactivator (Skrzypek et al., 2017). We expected that, if *MBF1* encodes a transcriptional coactivator, the product of this gene should have an impact on the regulation of gene expression in the cells. To address *MBF1*-dependent gene expression, we analyzed gene expression patterns between the *mbf1*Δ/Δ mutant and the parent wild type strain. The goal of these experiments was to understand which genes were regulated by Mbf1. We started with the simplest and most direct approach for this assessment, which is to compare gene expression between a wild type and an *mbf1*Δ/Δ mutant using *in vitro* cultures during logarithmic phase growth in complete rich medium (YEPD). Under these conditions, there were 253 genes upregulated and 260 genes downregulated in the *mbf1*Δ/Δ mutant as compared to the wild type (2-fold change threshold, adjusted *p* ≤ 0.05) (Supplementary File 1).

**Figure 1:**
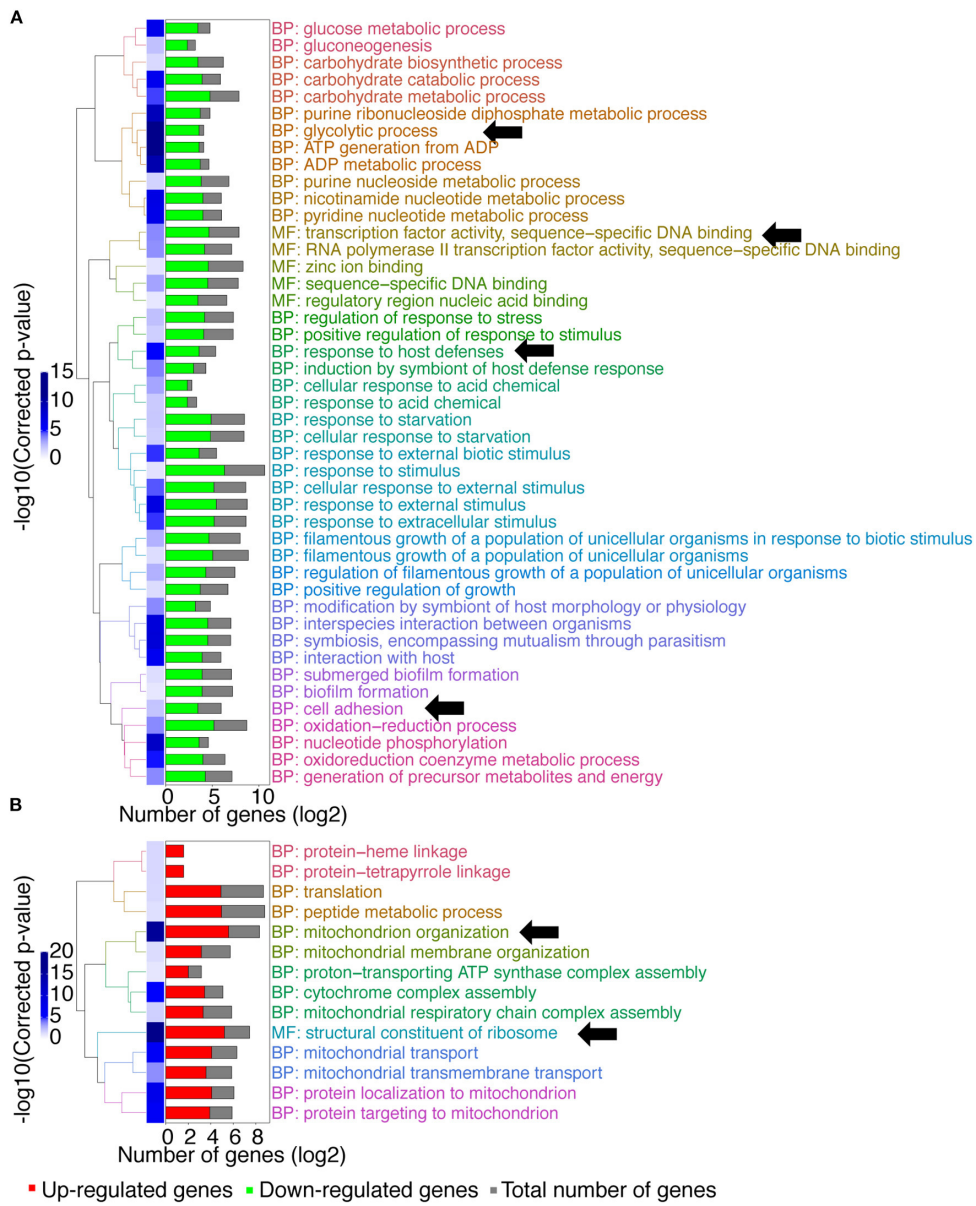
GO-terms enrichments in the *mbf1*Δ/Δ mutant compared to wild type when grown *in vitro* in YEPD at logarithmic phase. Hierarchical clustering of GO term is shown. **(A)** GO-terms enrichments of down-regulated genes compared to wild type. **(B)** GO-terms enrichments of up-regulated genes compared to wild type. The complete list of GO-terms can be found in Supplementary File 1. For simplification, all redundant GO-terms as well as too general GO-terms were removed. GO-terms discussed in the text are shown by arrows. Only genes with a fold-change ≥ 2 and an adjusted *p* ≤ 0.05 were included in the GO term analysis. GO term analysis performed with Candida Genome Database GO Term Finder (http://candidagenome.org/cgi-bin/GO/goTermFinder).

A gene ontology (GO) term enrichment analysis was first performed on the obtained data (Figure 1). Interestingly, downregulated genes were enriched for genes with transcription factor activity (up to 25 genes, Supplementary File 1; molecular function (MF): transcription factor activity, sequence–specific DNA binding, Figure 1A). Given that *MBF1* plays a potential role as a transcriptional coactivator, one could have expected such a result. For example, the transcription factors genes *BRG1, ROB1*, or *EFG1* that are involved in biofilm formation were downregulated in the *mbf1*Δ/Δ mutant. These genes target themselves other transcription factors such as *NRG1, CRZ2*, and *CUP9*, which are also downregulated in the *mbf1*Δ/Δ mutant (Nobile et al., 2012). Another important category of downregulated genes was those involved in glycolytic processes (up to 12 genes, Supplementary File 1; biological process (BP): glycolytic process, Figure 1A) including, for example, *ENO1, TDH3*, and *PGK1*. This signature signifies a change in metabolic adaptation of *C. albicans* when *MBF1* is absent. In addition, genes responding to host conditions were downregulated, such as *ENO1, SSK1*, or *CDC19* (up to 12 genes, Supplementary File 1; BP: response to host defenses, Figure 1A), as were genes implicated in the response to starvation, such as *ZRT2, FET34, HAP43*, or *SHA3* (up to 22 genes, Supplementary File 1; BP: response to starvation, Figure 1A). Genes implicated in adhesion (e.g., *ALS2, ALS4, ALS9, SAP9, ECM331*) (up to 14 genes, Supplementary File 1; BP: cell adhesion, Figure 1A) were also expressed at lower levels in the *mbf1*Δ/Δ mutant. The processes mentioned here are all known to be regulated by *C. albicans* during infection (Amorim-Vaz et al., 2015b). The fact that the *mbf1*Δ/Δ mutant cells express these genes at lower levels could partially explain the defect of virulence of the *mbf1*Δ/Δ mutant observed previously (Amorim-Vaz et al., 2015a). Regarding processes upregulated in the absence of *MBF1*, we observed genes involved in mitochondrial organization and function (e.g., *TIM21, TOM20*, or *MRP20*) (up to 48 genes, Supplementary File 1; BP: mitochondrion organization, Figure 1B), as well as ribosomal organization and translation (e.g., *RPL38, RPS30*, or *MRPL10*) (up to 37 genes, Supplementary File 1; MF: structural constituent of ribosome, Figure 1B). These observations could have different explanations. It is possible that the genes involved in these processes are repressed in the presence of Mbf1. Alternatively, alteration of mitochondrial functions could be a compensatory mechanism to metabolic changes in the *mbf1*Δ/Δ mutant (reduced glycolysis and ATP generation). It is interesting to observe that *MIG1*, a gene encoding a transcription factor that represses respiratory metabolism in *C. albicans* (Lagree et al., 2020) is repressed in the *mbf1*Δ/Δ mutant, which could be responsible, at least partially, for the increased expression of genes involved in mitochondrial function.

**Figure 2:**
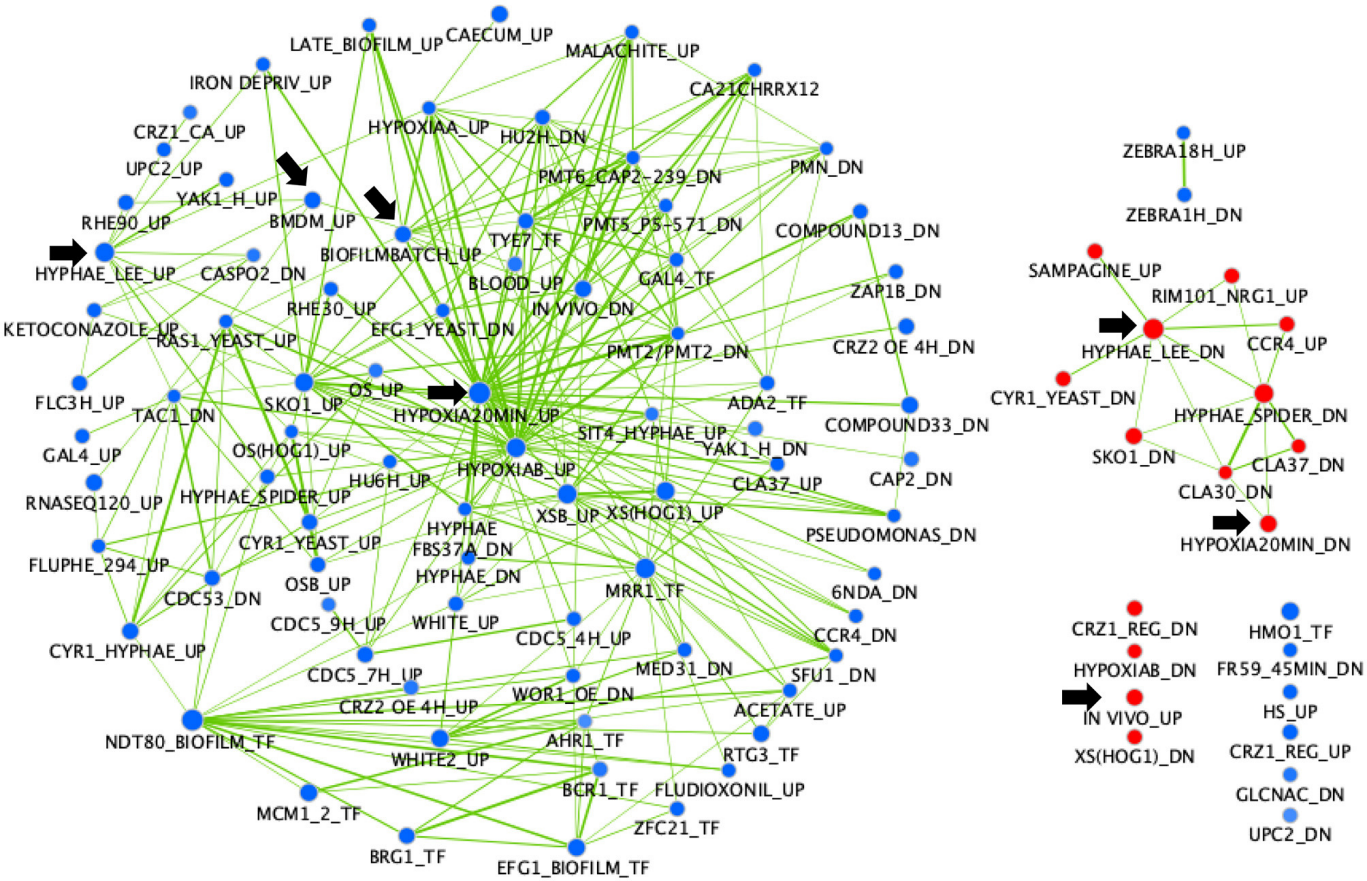
GSEA of *C. albicans* genes regulated in the *mbf1*Δ/Δ mutant relative to the wild type strain when grown *in vitro* in YEPD at logarithmic phase. The gene list was produced from data in Supplementary File 1 (containing the list of genes differentially regulated in the mutant, with fold change ≥ 2 (up or down) and adjusted *p* ≤ 0.05 in the *mbf1*Δ/Δ mutant relative to the wild type) and Supplementary File 2 (containing the gene sets used for comparison). The analysis was then performed as described in Amorim-Vaz et al. (2015b). Red nodes represent enriched gene lists among the upregulated genes. Blue nodes represent enriched gene lists among the downregulated genes. Nodes are connected by edges when overlaps exist between nodes. The size of nodes reflects the total number of genes that are connected by edges to neighboring nodes. Edge thickness reflects the level of confidence between nodes. Black arrows indicate nodes that are discussed in the text.

A gene set enrichment analysis (GSEA) was performed on the same transcriptional data (Figure 2). This analysis is useful to show what other conditions result in transcriptional profiles overlapping the profile of the *mbf1*Δ/Δ mutant. Interestingly, this analysis revealed overlaps of downregulated genes in the absence of *MBF1* with those that were upregulated in several environments linked to virulence. For example, there was a significant overlap (node “IN VIVO_UP,” 64 genes) between genes upregulated during infection of *C. albicans* in different models (mouse, *Galleria mellonella*) (Amorim-Vaz et al., 2015b) or in co-culture with bone marrow-derived macrophages (node “BMDM_UP”, 61 genes) (Marcil et al., 2008) and genes downregulated in the *mbf1*Δ/Δ mutant (Supplementary File 2). Similarly, genes that were upregulated during hyphal formation in Lee medium (node “HYPHAE_LEE_UP,” 98 genes) (Nantel et al., 2002), biofilm formation (node “BIOFILMBATCH_UP,” 58 genes) (Sellam et al., 2009) or response to hypoxia (node “HYPOXIA20MIN_UP,” 127 genes) (Sellam et al., 2014) were rather downregulated in the absence of *MBF1* (Supplementary File 2). The opposite was also true, as the overlap between genes downregulated in hyphal-inducing conditions (node “HYPHAE_LEE_DN,” 113 genes) (Nantel et al., 2002) and during hypoxia (node “HYPOXIA20MIN_DN,” 65 genes) (Sellam et al., 2014) and genes upregulated in the *mbf1*Δ/Δ mutant was significant (Supplementary File 2).

### *MBF1*-Dependent Gene Expression *in vivo*

The study of genes regulated by Mbf1 *in vitro* provided interesting results, however it was also important to analyze these differences *in vivo*. This was motivated by the following rationale: (i) the *mbf1*Δ/Δ mutant shows a decrease of virulence during mouse infections and thus could help the identification of *MBF1-*dependent genes under these conditions; (ii) it is well-established that regulatory circuits of transcription can differ considerably between *in vitro* and *in vivo* conditions (Kumamoto, 2008; Fanning et al., 2012).

We attempted to perform RNA sequencing on RNA extracted from mouse kidneys infected with wild type *C. albicans* or the *mbf1*Δ/Δ mutant. In Amorim-Vaz et al. (2015b), this type of experiment was performed for kidneys infected with a wild type strain only, RNA extracted at 16 and 48 h post-infection (p.i.) and using the customized SureSelect RNA enrichment method to obtain a high coverage of fungal transcripts. The same process was attempted for the study of *MBF1*-dependent transcription *in vivo*. RNA-seq of the samples infected with the *mbf1*Δ/Δ mutant at 16 h p.i. resulted in too few reads mapped to *C. albicans*, impairing a reliable analysis of the data [Supplementary Table 4 (Data Sheet 6.docx)]. This was understandable considering that the *mbf1*Δ/Δ mutant generates lower fungal burdens in the kidneys than the wild type strain (Amorim-Vaz et al., 2015a). The 16 h p.i. time point was then excluded from further analysis.

**Figure 3:**
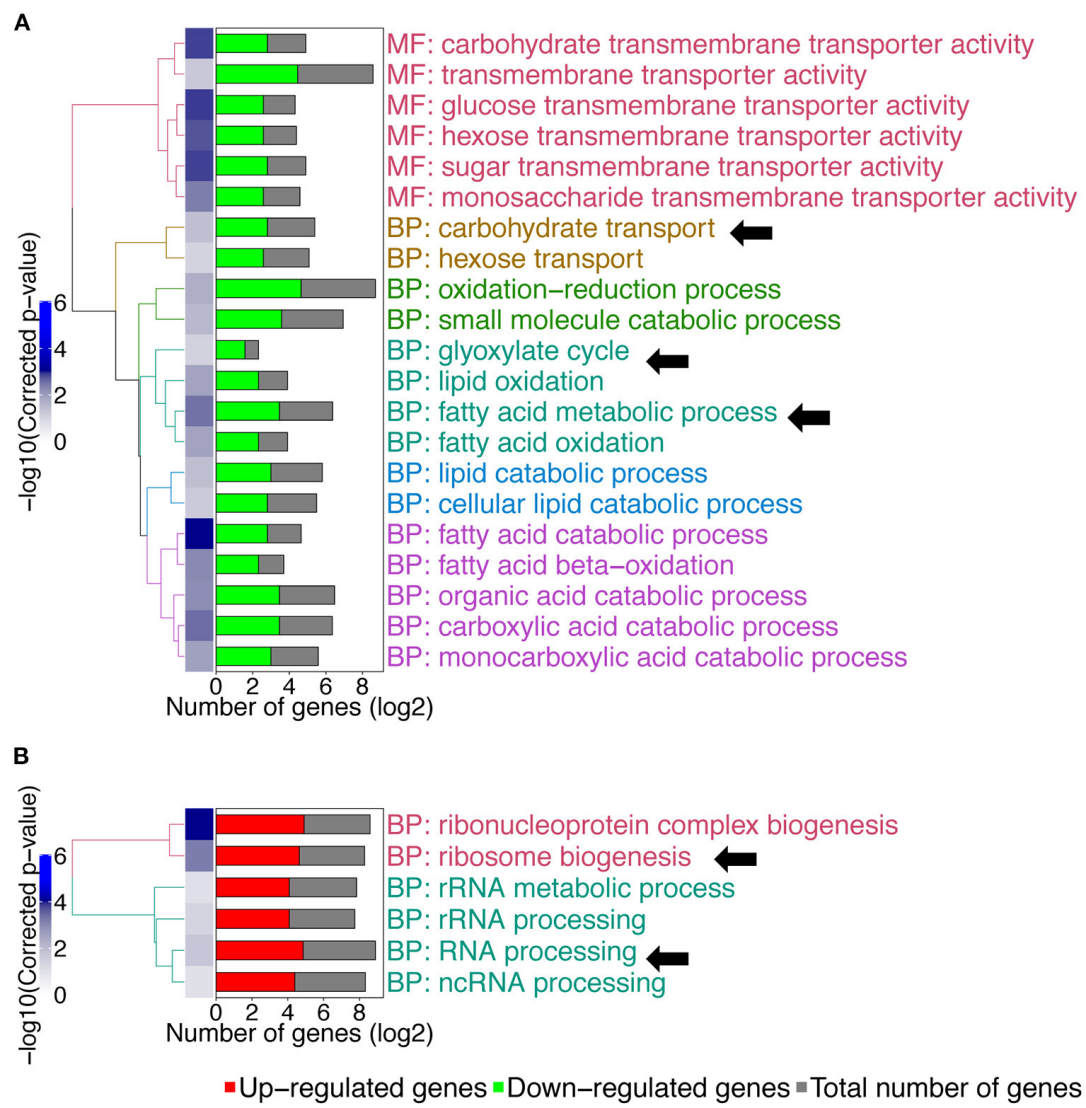
GO-terms enrichments in the *mbf1*Δ/Δ mutant compared to wild type *in vivo*. Hierarchical clustering of GO term is shown. **(A)** GO-terms enrichments of down-regulated genes compared to wild type. **(B)** GO-terms enrichments of up-regulated genes compared to wild type. The complete list of GO-terms can be found in Supplementary File 3. For simplification, all redundant GO-terms as well as too general GO-terms were removed. GO-terms discussed in the text are shown by arrows. Only genes with a fold change ≥ 2 and a raw *p* ≤ 0.05 were included in the GO term analysis. GO term analysis performed with Candida Genome Database GO Term Finder (http://candidagenome.org/cgi-bin/GO/goTermFinder).

We then compared gene expression profile of the *C. albicans mbf1*Δ/Δ mutant to the wild type during systemic infection of mouse kidneys at 48 h p.i. (Supplementary File 3). To enlarge the number of genes that could be included in the study, the stringency of the statistical analysis was reduced with a cut-off of 0.05 on the raw *p*-values, instead of the adjusted *p*-values. The raw *p*-value indicates the statistical significance for the fold-change of a given gene. However, when making multiple comparisons the chance of obtaining a significant difference increases, thus leading to an increase in the possibility of false positive results. The adjusted *p*-value takes this into account and increases stringency. Keeping this limitation in mind and applying a raw *p* ≤ 0.05 threshold and maintaining a 2-fold threshold in gene expression level, 206 genes were upregulated and 187 genes were downregulated in the *mbf1*Δ/Δ mutant as compared to the wild type (Supplementary File 3). A GO-term enrichment analysis indicated that genes involved in fatty acid metabolism, carbohydrate transport and the glyoxylate cycle were downregulated *in vivo* in the mutant relative to the wild type (GO terms indicated by arrows in Figure 3A). These are processes usually activated by *C. albicans* during infection (Amorim-Vaz et al., 2015b). These processes were upregulated in the *mbf1*Δ/Δ mutant *in vivo* as compared to the *mbf1*Δ/Δ mutant *in vitro* (Supplementary File 4). However, the level of upregulation was lower than in the wild type strain, suggesting a diminished capacity of the mutant to adapt to an environment with limited carbohydrate availability. This is in line with the observation that the *mbf1*Δ/Δ mutant had reduced expression of genes involved in response to nutrient levels and starvation *in vitro* (relative to the wild type) (Figure 1A). Regarding genes upregulated in the *mbf1*Δ/Δ mutant as compared to the wild type during infection, the GO terms ribosome biogenesis and RNA processing were enriched (GO terms indicated by arrows Figure 3B). This could indicate that protein synthesis is more active in the mutant than in wild type *in vivo*. Again, this is a process usually downregulated during infection with the wild type *C. albicans* (Amorim-Vaz et al., 2015b), presumed to be an adaptation to the low amount of amino acids available. Even though these processes were downregulated in the *mbf1*Δ/Δ mutant *in vivo* as compared to the *mbf1*Δ/Δ mutant *in vitro* (Supplementary File 4), they were still more active in the mutant than in the wild type in mouse kidneys. This is aligned with the fact that translation and peptide synthesis are upregulated in the mutant as compared to the wild type when both are grown *in vitro* (Figure 1B). Together, these observations suggest a role for *MBF1* in the adaptation of *C. albicans* metabolism to the conditions encountered during infection.

### Interaction of Mbf1 With the Transcription Machinery

According to predictions based on protein sequence, *MBF1* may encode a transcriptional coactivator. Therefore, it is expected to physically interact with other transcription factors. Looking at the function of homolog proteins in closely related species can provide hints about the functions of Mbf1 in *C. albicans*. In *S. cerevisiae*, Mbf1 was shown to recruit the TATA-binding protein (TBP) and to bridge the latter to transcription factors, allowing the initiation of transcription of genes regulated by these transcription factors (Takemaru et al., 1997).

The transcriptional analysis of the *mbf1*Δ/Δ mutant provided some clues about processes in which Mbf1 might be implicated. For instance, the data suggested a connection of *MBF1* with the adaptation of metabolism to the nutrient levels in the environment. In *C. albicans*, protein synthesis was shown to be repressed during infection (Amorim-Vaz et al., 2015b), presumably due to the limited availability of nitrogen sources in the environment. However, this repression was partly dependent on *MBF1* (as shown above). The master regulator of amino acid and protein synthesis in *C. albicans* is Gcn4. This transcription factor coordinates both metabolic and morphogenetic responses to amino acid starvation in *C. albicans*. It activates amino acid biosynthetic genes while repressing de-novo protein biosynthesis, and is necessary for resistance to amino acid starvation (Tripathi et al., 2002). Interestingly, in *S. cerevisiae*, Gcn4 action was shown to be mediated by Mbf1 bridging to TBP (Takemaru et al., 1998). This raised the hypothesis that Mbf1 could be a coactivator of Gcn4 in *C. albicans*, similar to what is known from *S. cerevisiae*.

### Contribution of *MBF1* to Resistance to Amino Acid Starvation

To verify this hypothesis, we started by investigating the effect of Mbf1 on resistance to amino acid starvation. If indeed the function of Gcn4 in *C. albicans* is dependent on Mbf1, then Mbf1 is expected to be necessary for resistance to amino acid starvation. A *gcn4*Δ/Δ mutant was previously shown to have defective growth on minimal media lacking only one amino acid, histidine (Tripathi et al., 2002). Therefore, the *mbf1*Δ/Δ mutant was spotted on minimal medium (YNB) either supplemented with all amino acids (YNB + CSM), all amino acids except histidine (YNB -his), or YNB -his supplemented with 5 mM of 3-AT. This compound is a competitive inhibitor of the *HIS3* gene product, a gene involved in the biosynthesis of histidine (Brennan and Struhl, 1980). In its presence, the amino acid starvation phenotypes are accentuated, and growth of a *gcn4*Δ/Δ mutant was strongly defective in its presence (Tripathi et al., 2002).

**Figure 4:**
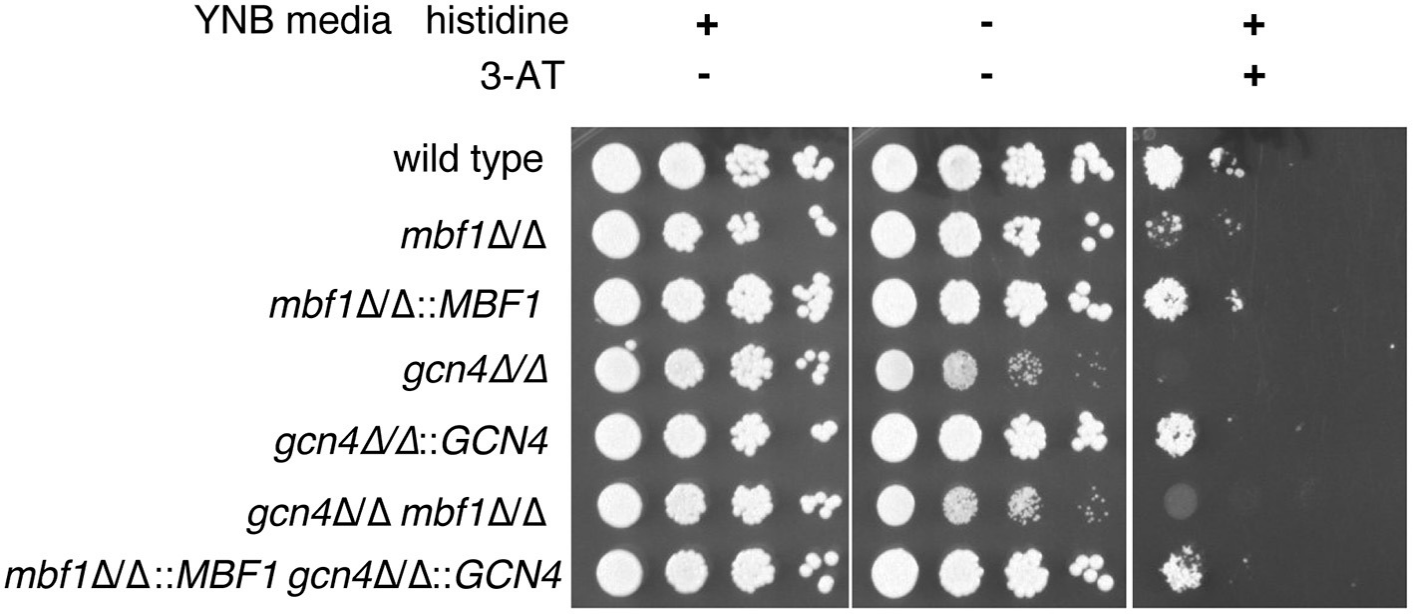
Assessment of the influence of *MBF1* on *C. albicans* resistance to amino acid starvation on solid media. Strains were grown in liquid media lacking histidine before serial 10-fold dilutions of each strain were spotted on YNB + CSM, YNB -his is or YNB -his + 3-AT (5 mM). Plates were incubated at 35°C and pictures taken 24 to 72 h later. Representative results are shown. Strain designations: Wild type—SC5314; *mbf1*Δ/Δ–ACY367; *mbf1*Δ/Δ::*MBF1*—SVY24; *gcn4*Δ/Δ–DSY4843; *gcn4*Δ/Δ::*GCN4*—DSY4925; *gcn4*Δ/Δ, *mbf1*Δ/Δ–DSY4917; *gcn4*Δ/Δ::*GCN4, mbf1*Δ/Δ::*MBF1*—DSY4926.

As shown in Figure 4, all strains grew alike in medium supplemented with all amino acids (left panel). The phenotype of the *gcn4*Δ/Δ mutant previously described by others (Tripathi et al., 2002) was reproduced with the mutant produced here. This mutant showed a defective growth in the absence of histidine as compared to the isogenic wild type strain and to the *GCN4* revertant strain (*gcn4*Δ/Δ::*GCN4*) (central panel). As expected, this phenotype was exacerbated with the addition of 3-AT to the medium (right panel). No obvious phenotype was visible for the *mbf1*Δ/Δ mutant in the absence of histidine. However, its growth was clearly affected as compared to the wild type in the presence of 3-AT (5 mM), although not as strongly as the *gcn4*Δ/Δ mutant. A similar trend was observed with the addition of 2.5 mM 3-AT [Supplementary Figure 2 (Data Sheet 7.docx)]. A mutant lacking both genes (*gcn4*Δ/Δ, *mbf1*Δ/Δ) was also tested. Interestingly, its phenotype seemed to be intermediate between the two individual mutants, an observation that was reproducible between experiments. All phenotypes for the mutants were reverted by reintroducing the corresponding wild type alleles at their native loci. These results suggest that *MBF1* is implicated in the response to amino acid starvation response, as is *GCN4*, although they do not prove a direct interaction between the encoded proteins.

### *MBF1*-Dependent Gene Expression During Amino Acid Starvation

Given that Mbf1 might be involved in the starvation response, it was interesting to verify whether this involvement was observed at the level of gene regulation. As mentioned before, it is well-established that Gcn4 plays a central role in nitrogen starvation response, activating the expression of different genes including genes involved in amino acid biosynthesis. Therefore, we decided to analyze gene regulation of *C. albicans* during amino acid starvation and to compare the wild type, the *mbf1*Δ/Δ mutant and the *gcn4*Δ/Δ mutant. The three strains were cultured in liquid YNB + CSM (control condition), YNB -his or YNB -his + 3-AT (1 mM) (starvation conditions). A low concentration of 3-AT was used (1 mM) in order to elicit a starvation response while still allowing growth in order to yield sufficient amounts of RNA. RNA was extracted and used for RNA sequencing. With the data obtained, hierarchical clustering and principal component analysis were performed [Supplementary Figure 3 (Data Sheet 7.docx)]. RNA seq data were validated by qPCR for a subset of regulated genes [Supplementary Figure 4 (Data Sheet 7.docx)]. During growth in complete medium, wild type and *mbf1*Δ/Δ mutant clustered closely together, separately from the *gcn4*Δ/Δ mutant. When histidine was absent from the medium, wild type and *mbf1*Δ/Δ mutant still clustered closely together, and the distance to the *gcn4*Δ/Δ mutant increased. With the addition of 3-AT, the data shows that the *mbf1*Δ/Δ mutant no longer clustered with the wild type but was closer to the *gcn4*Δ/Δ mutant. Principal component analysis indicated that the *mbf1*Δ/Δ mutant only responded differently from the wild type in the 3-AT condition. Therefore, we calculated differential gene expression for each strain for the starvation condition (YNB -his + 3-AT culture :starvation) as compared to the control condition (YNB + CSM culture: control) (Supplementary File 5):


$$\begin{array}{l} WT_{\text{starvation}}/WT_{\text{control}}=FC_{WT}\\ {mbf1\Delta \Delta }_{\text{starvation}}/{mbf1\Delta \Delta }_{\text{control}}\;=FC_{mbf1}\\ {gcn4\Delta \Delta }_{\text{starvation}}/{gcn4\Delta \Delta }_{\text{control}}\;=FC_{gcn4} \end{array}$$

**Figure 5:**
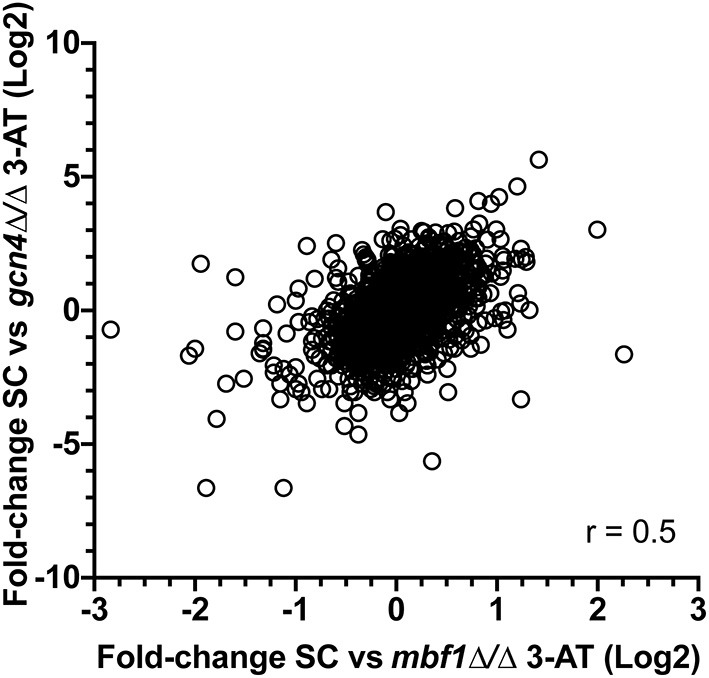
Correlation between transcriptional responses to starvation in the two mutants. For each regulated gene (2,264 genes with adjusted *p* ≤ 0.05 in FC_WT_, FC_*mbf*1_, and FC_*gcn*4_, see Supplementary File 5), the ratios between expression fold-change in each mutant as compared to wild type (FC_WT_/FC_*gcn*4_ and FC_WT_/FC_*mbf*1_) is calculated. Values were plotted and Spearman correlation was calculated using GraphPad Prism software (version 8.4.3). *r* = 0.5, *p* ≤ 0.0001.

We next calculated for each regulated gene the ratios between expression fold-change in each mutant as compared to wild type (FC_WT_/FC_*gcn*4_ and FC_WT_/FC_*mbf*1_). This transformation revealed how a gene was regulated in the mutants as compared to the wild type (Supplementary File 5). A correlation between FC_WT_/FC_*gcn*4_ and FC_WT_/FC_*mbf*1_ (Figure 5) showed that there was a general concordance in the direction of the ratio between the two mutants and that this correlation was statistically significant (Spearman *r* = 0.5, *p* ≤ 0.0001).

**Figure 6:**
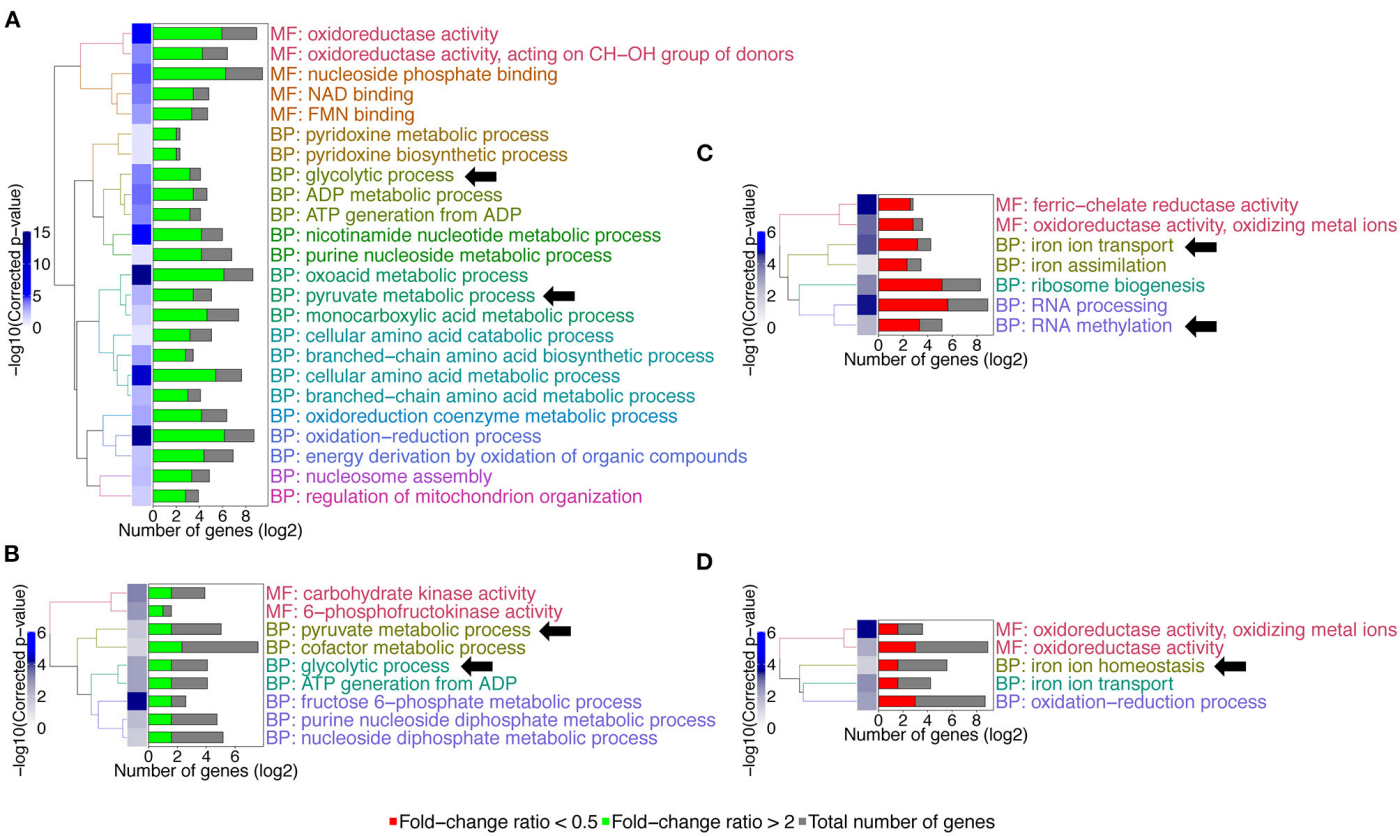
GO-terms enrichments in the *gcn4*Δ/Δ and *mbf1*Δ/Δ mutants compared to wild type in response to starvation. Hierarchical clustering of GO term is shown. **(A)** GO-terms enrichments of up-regulated genes between *gcn4*Δ/Δ mutant and the wild type (FC_WT_/FC_gcn4_ > 2). **(B)** GO-terms enrichments of down-regulated genes between *gcn4*Δ/Δ mutant and the wild type (FC_WT_/FC_gcn4_ < 0.5). **(C)** GO-terms enrichments of up-regulated genes between *mbf1*Δ/Δ mutant and the wild type (FC_WT_/FC_*mbf*1_> 2). **(D)** GO-terms enrichments of down-regulated genes between *mbf1*Δ/Δ mutant and the wild type (FC_WT_/FC_mbf1_ < 0.5). The complete list of GO-terms can be found in Supplementary File 5. For simplification, all redundant GO-terms as well as too general GO-terms were removed. GO-terms discussed in the text are shown by arrows. Only genes with a fold-change ≥ 2 and an adjusted *p* ≤ 0.05 were included in the GO term analysis. GO term analysis performed with Candida Genome Database GO Term Finder (http://candidagenome.org/cgi-bin/GO/goTermFinder).

The calculation of the above-mentioned ratios allowed a better comparison of the two strains. Four hundred and thirty-four genes (434) were at least 2-fold upregulated in the wild type than in the *gcn4*Δ/Δ mutant when comparing growth between starved (YNB -his +3-AT) and non-starved (YNB+CSM) conditions (FC_WT_/FC_*gcn*4_ ≥ 2). The reverse was true for 351 genes (FC_WT_/FC_*gcn*4_ ≤ 0.5). In the *mbf1*Δ/Δ mutant, the number of genes falling into these thresholds was 24 and 27 genes, respectively. A GO term enrichment analysis revealed that the group of 434 genes in the category “FC_WT_/FC_*gcn*4_ ≥ 2” was enriched in genes involved in amino acid metabolism, which were upregulated in the wild type during growth in starvation conditions, but downregulated in the *gcn4*Δ/Δ mutant. In the same category were the GO terms glycolysis and pyruvate metabolism (GO terms indicated by arrows Figure 6A). The genes involved in these processes were downregulated in the wild type, but further downregulated in the *gcn4*Δ/Δ mutant. Interestingly, the processes of glycolysis and pyruvate metabolism were also enriched among the genes under “FC_WT_/FC_*mbf*1_ ≥ 2,” but not amino acid metabolism (GO terms indicated by arrows in Figure 6B). However, a closer look at fold-change values for the genes included in “amino acid metabolism” revealed that these genes were regulated in a similar direction in both mutants, however the threshold FC_WT_/FC_*mbf*1_ > 2 was not reached in the *mbf1*Δ/Δ mutant (Supplementary File 5). On the other hand, RNA processing and RNA methylation, as well as iron transport and homeostasis, were enriched processes among the genes in the category FC_WT_/FC_*gcn*4_ < 0.5 (GO terms indicated by arrows in Figure 6C). The corresponding genes were generally downregulated in the wild type when comparing control and starvation conditions, but were upregulated in the *gcn4*Δ/Δ mutant, and thus were genes possibly repressed in the presence of Gcn4. Iron transport and homeostasis were also enriched among genes in the group FC_WT_/FC_*mbf*1_ < 0.5, but not RNA processing or methylation (Figure 6D).

Lastly, the proportion of genes with a binding site for Gcn4 in their promoters (1,000 bp upstream of start codon) was examined (Table 1). The binding site for *C. albicans* Gcn4 has the sequence “TGACTM” (with M being either A or C) (Gasch et al., 2004). Among the genes upregulated in the wild type strain during starvation, the proportion of genes with a Gcn4 binding site was around 81%. For comparison, the proportion of genes with a Gcn4 binding site in *C. albicans* (among all 5,548 genes expressed and detected by RNA-seq) was 54%. This reinforces the central role played by this transcription factor in the response to starvation, since most of the genes activated in this condition were regulated by Gcn4. In the *gcn4*Δ/Δ mutant, the proportion of the upregulated genes with a Gcn4 binding site dropped to 52%, similar to the basal level among all expressed genes, as expected. In the *mbf1*Δ/Δ mutant, this proportion is of 72%. A possible interpretation of these observations is that around 10% of the genes normally activated by Gcn4 in response to starvation are also dependent on Mbf1.

**Table 1:** Percentage of genes with Gcn4-binding sites in the 1,000 bp upstream start codon.

	**Wild type**	***gcn4*Δ/Δ**	***mbf1*Δ/Δ**	**All *C. albicans* genes** ^**c**^
Upregulated in starvation^a^	81%	52%	72%	54%
Downregulated in starvation^b^	48%	55%	48%	

a *Fold-change in starvation relative to control ≥ 2*.

b *Fold-change in starvation relative to control ≤ 0.5*.

c *5,548 genes expressed and detected by RNA-seq with at least one count per million in at least one condition*.

### Cellular Localization of Mbf1

**Figure 7:**
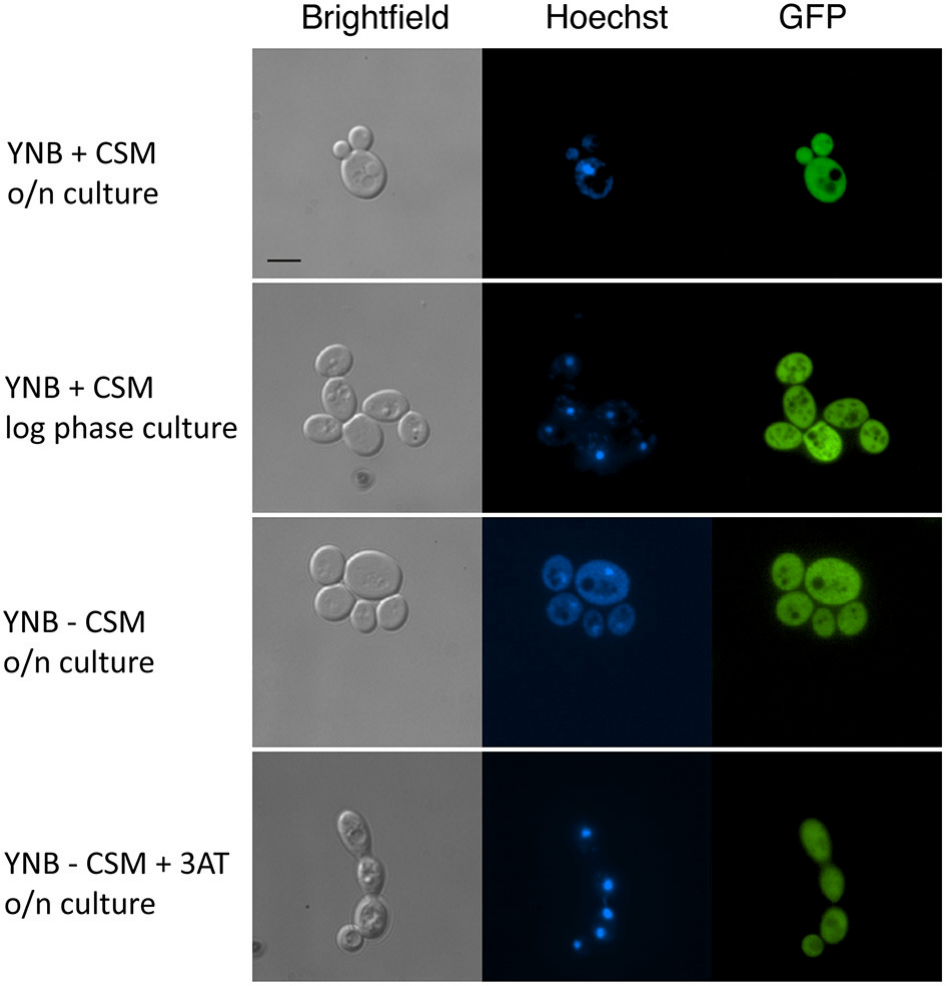
Cellular localization of Mbf1-GFP. Pictures show brightfield, Hoechst fluorescence (DNA staining, blue), GFP (green). *MBF1-GFP* tagged strain (SVY26) were grown overnight (O/N) in YNB supplemented or not with amino acids (+ CSM or – CSM, respectively). An aliquot of the O/N culture was diluted and grown for 3 h in fresh media (YNB + CSM or YNB – CSM + 3-AT 2 mM). Scale bar: 5 μm (applicable to all pictures).

Since *MBF1* encodes a putative transcriptional co-activator, it was reasonable to predict it to be localized in the nucleus. This is also the expectation according to an online platform for prediction of protein features, which localizes Mbf1 in the nucleus (Yachdav et al., 2014). In addition, *MBF1* is annotated in Candida Genome Database as containing a Lambda repressor-like, DNA-binding domain (IPR010982). To verify this, a strain expressing an Mbf1-GFP fusion protein was constructed with GFP fused at the C-terminal of Mbf1. The functionality of the fused protein was verified by spotting the new strain (SVY26) on YNB -his with 5 mM 3-AT after growth for 3 h in liquid YNB -his. The GFP-tagged strain showed levels of growth similar to the wild type, unlike the *MBF1* null mutant thus confirming that Mbf1 was functional in GFP tagged strain [Supplementary Figure 5 (Data Sheet 7.docx)]. When this strain was grown in complete medium (YNB + CSM), Mbf1 appeared not to be localized in a specific organelle both in logarithmic and stationary phase cells (Figure 7). Fluorescence profile was not different in cells grown under amino acid starvation (Figure 7). The absence of a specific localization (such as the nucleus) indicate that Mbf1 may also interact with non-nuclear proteins. Mbf1 cytoplasmic localization is known from other localization studies due to its interaction with several other cytoplasmic proteins (for example heat shock proteins, ribosomal proteins) (Wang et al., 2018).

### Interaction of Mbf1 With Gcn4 Using Fluorescence Reporters

**Figure 8:**
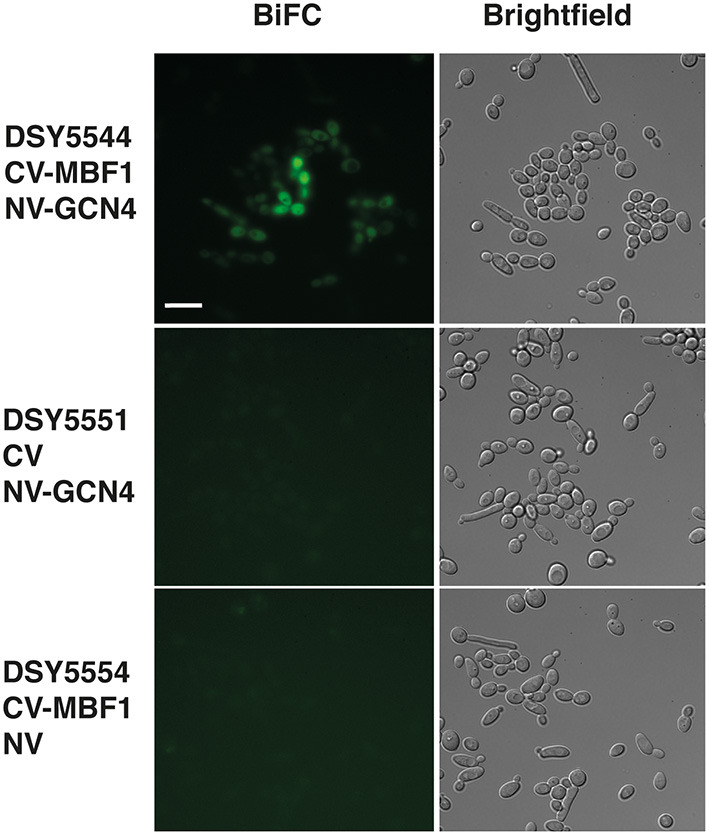
Interaction of Mbf1 with GcN4. Pictures show epifluorescence (left, bimolecular fluorescence complementation, BiFC) and brightfield microscopy under differential interference contrast (right, Brightfield). Cells were grown as described under Material and Methods and grown for 3–4 h in YNB +his but without methionine and cysteine to induce fusion proteins. Scale bar: 10 μm (applicable to all pictures). *Candida albicans* strains used are shown at the left side with the types of fusion protein. CV-MBF1, N-terminal tagging of the C-terminal part of yEmVenus; CV, C-terminal part of yEmVenus; NV-GCN4, N-terminal tagging of the N-terminal part of yEmVenus; NV, N-terminal part of yEmVenus.

Our data suggest that Mbf1 and Gcn4 may participate to amino acid starvation. Other data from *S. cerevisiae* showed that Mbf1 physically interacted with Gcn4 (Takemaru et al., 1998). However, these data were performed with purified proteins and interaction was addressed essentially *in vitro*. We aimed to probe the interaction between Mbf1 and Gcn4 in the context of the natural cell environment in *C. albicans*. A bimolecular fluorescence complementation (BiFC) assay has been established in *C. albicans* that allows the study of protein interactions *in vivo*. In this assay, proteins are tagged with yeast enhanced monomeric Venus (yEmVenus) fragments, which upon interaction reconstitutes a functional yEmVenus and emission of fluorescence. We therefore tagged Mbf1 and Gcn4 with these fragments and assessed fluorescence emission. As shown in Figure 8 (top panel), the presence of both N-terminal tagged proteins with each the N- and C-terminal parts of bimolecular complementation of yEmVenus generated fluorescence in *C. albicans*. This was not the case when one of the fusion proteins was replaced by controls, i.e., presence of the unfused N- and C-terminal parts yEmVenus (Figure 8, bottom panels). Fluorescence was mainly localized to the cytoplasm as was the case for Mbf1-GFP (Figure 8). Our data therefore reinforce the idea that Mbf1 and Gcn4 interact in *C. albicans*.

### Contribution of *GCN4* to Virulence

**Figure 9:**
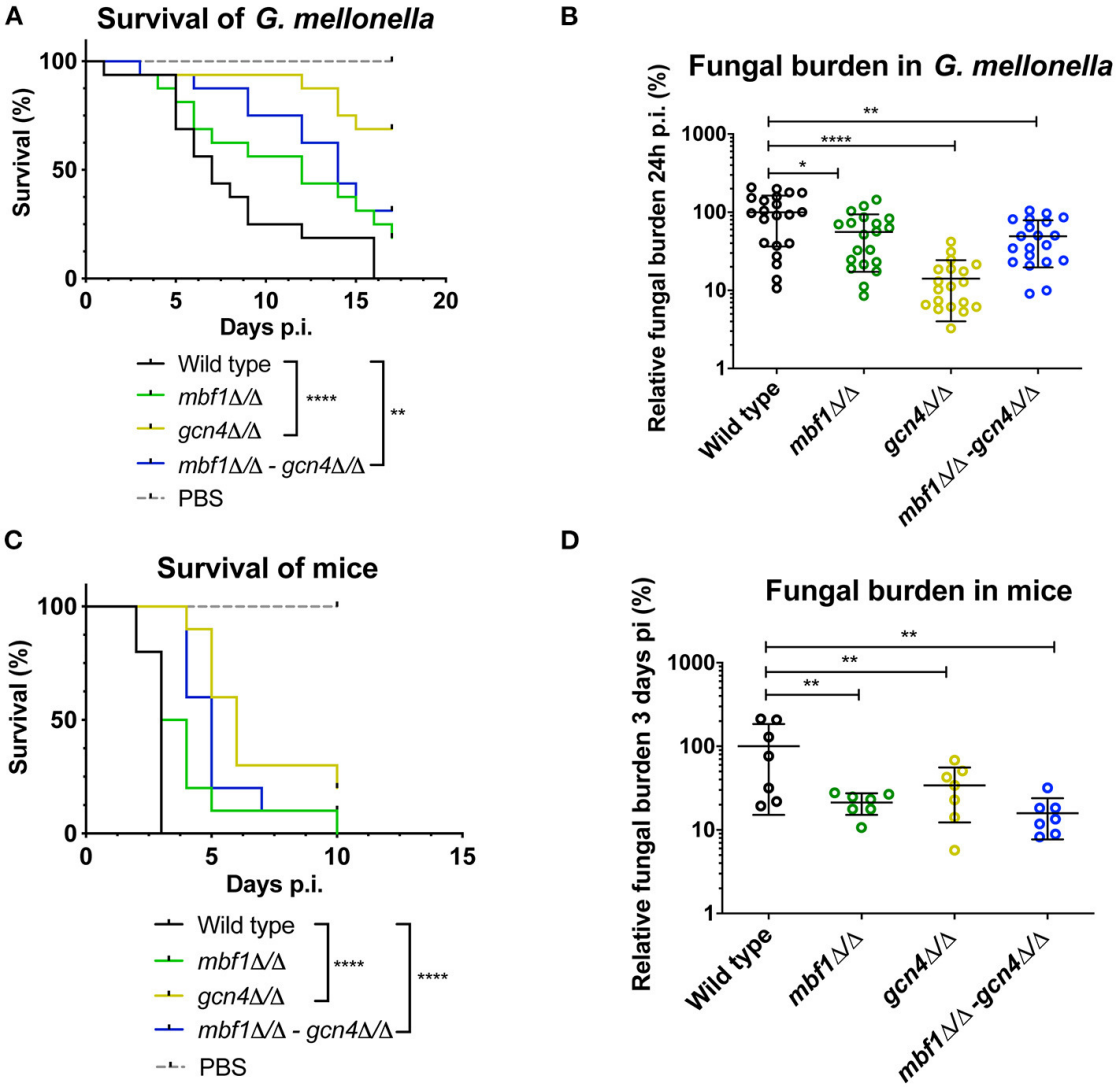
Survival rates and fungal burdens in larvae of *G. mellonella* **(A,B)** and mice **(C,D)** with *mbf1*Δ/Δ, *gcn4*Δ/Δ and double mutant. For survival curves, larvae or mice were injected with 5 × 10^5^ cells of the indicated strains and monitored daily. Curves are representative of at least two independent experiments. Statistical analysis was performed using the log-rank test. For fungal burden assays, fungal burdens were expressed as a percentage relative to the mean of the wild type group in the same experiment. All experiments were pooled. The mean and standard error of each group is indicated with horizontal black bars. Statistical analyses were performed using a ROUT analysis to remove outliers (Motulsky and Brown, 2006), followed by a Mann–Whitney test to assess CFU differences relative to the wild type strain. ^*^p < 0.05, ^**^p < 0.01, ^****^p < 0.0001.

**Figure 10:**
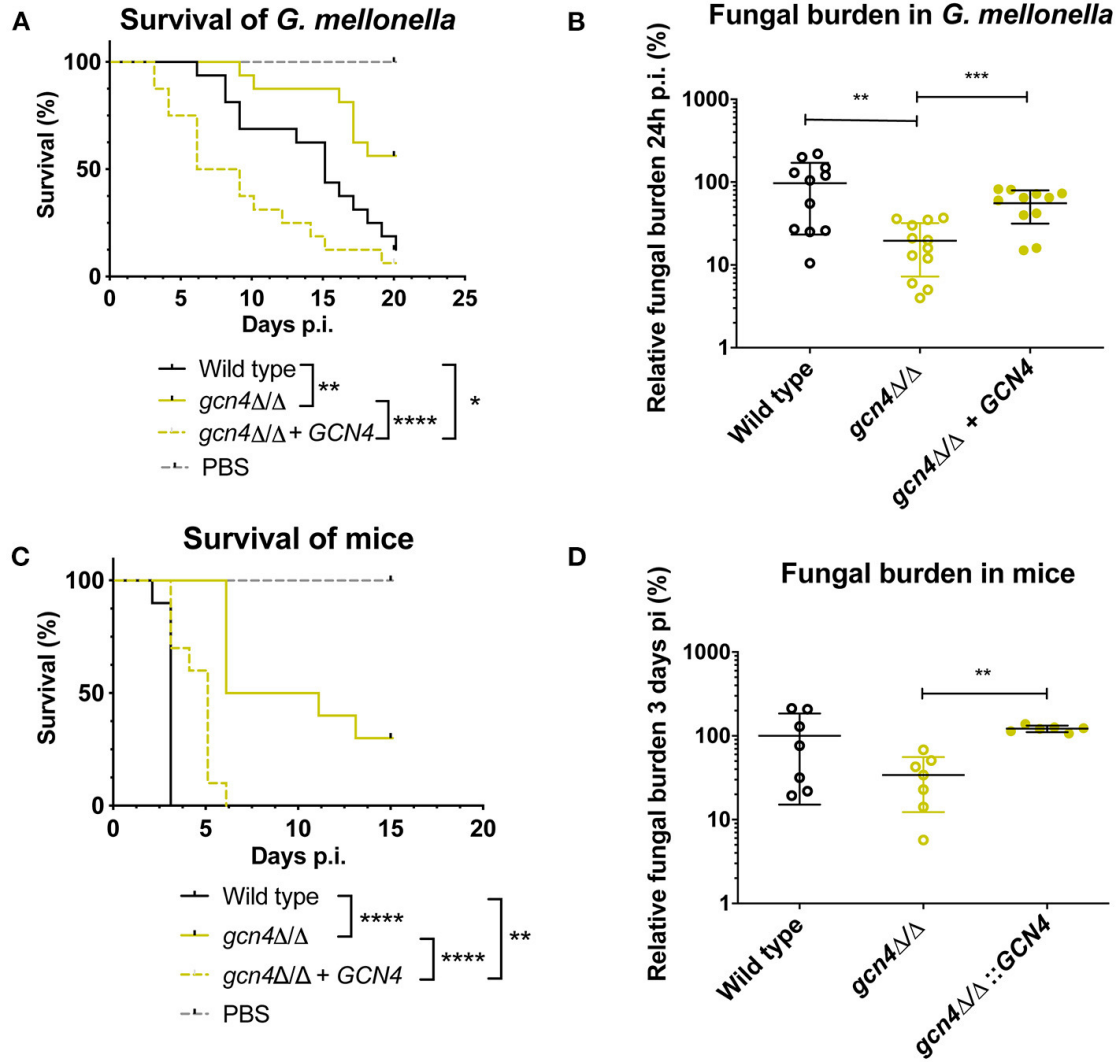
Survival rates and fungal burdens with *gcn4*Δ/Δ mutant and revertant strain in larvae of *G. mellonella* **(A,B)** and mice **(C,D)**. For survival curves, larvae or mice were injected with 5 × 10^5^ cells of the indicated strains and monitored daily. Curves are representative of at least two independent experiments. Statistical analysis was performed using the log-rank test. For fungal burden assays, fungal burdens were expressed as a percentage relative to the mean of the wild type group in the same experiment. All experiments were pooled. The mean and standard error of each group is indicated with horizontal black bars. Statistical analyses were performed using a ROUT analysis to remove outliers (Motulsky and Brown, 2006), followed by a Mann–Whitney test to assess CFU differences relative to the wild type strain. ^*^p < 0.05, ^**^p < 0.01, ^***^p < 0.001, ^****^p < 0.0001.

We described earlier that the absence of *MBF1* in *C. albicans* resulted in decreased virulence in animal models (Amorim-Vaz et al., 2015a). If indeed reduced virulence of the *mbf1*Δ/Δ mutant is related to the defective adaptation of the metabolism to the nutrient levels *in vivo*, then a *gcn4*Δ/Δ mutant would also be expected to be attenuated in virulence. However, the literature on this subject is controversial. In Brand et al. (2004), a *gcn4*Δ/Δ mutant was found to reduce virulence in a mouse model of systemic infection, but this defect was maintained even after reintroduction of the wild type allele. This unexpected result was found to be due to the ectopic expression of the *URA3* gene (the selective marker used for mutant construction) at the *GCN4* locus. When *URA3* was introduced at the *RPS10* locus, the *gcn4*Δ/Δ mutant showed similar virulence to the isogenic wild type strain, as did the corresponding revertant strain. This study highlighted the impact of *URA3* expression levels on virulence, stressing the need to work with alternative and neutral selection markers for yeast genetic constructions. In addition, and given their results, the authors concluded that *GCN4* was not necessary for *C. albicans* virulence. The mutants used in the present study were constructed using *SAT1* as a selective marker. *SAT1* gene product provides resistance to the antimicrobial nourseothricin and is removed from the genome of the final mutant or revertant strains. The *gcn4*Δ/Δ and *mbf1*Δ/Δ mutants and double mutant, along with the respective revertant strains and their isogenic wild type strain (SC5314) were used in systemic infection of mice and of *Galleria mellonella*. We evaluated both the survival curves of the animals and the CFU counts (in the mice kidneys or in the entire *Galleria* larvae) originated by each strain. The results are presented in Figure 9. The *mbf1*Δ/Δ mutant exhibited decreased virulence in both mice and *G. mellonella*, as shown by the prolongation in animal survival represented (Figures 9A,C). Although the reduced virulence phenotype of the *mbf1*Δ/Δ mutant did not reach statistical significance, a clear tendency was observed and reproduced across experiments. Furthermore, these observations reproduce the results obtained in Amorim-Vaz et al. (2015a). Statistical significance was however reached between wild type and *mbf1*Δ/Δ mutant when considering fungal burden obtained by each type of strain (Figures 9B,D). The *gcn4*Δ/Δ mutant showed a very clear and statistically significant reduction of virulence and fungal burdens in both animal models. Interestingly, the double *gcn4*Δ/Δ *mbf1*Δ/Δ mutant reproducibly displayed an intermediate virulence phenotype between the two individual mutants in both animal models. According to these results, *GCN4* is indeed necessary for virulence, not only in mice but also in *G. mellonella*, contrary to what was previously published (Brand et al., 2004). To confirm that the results observed here were due to the absence of *GCN4* and not to some other artifacts, the *GCN4* revertant strain was also tested and results are shown in Figure 10. By reintroducing one *GCN4* allele in the *gcn4*Δ/Δ mutant, the phenotypes of virulence and low fungal burden were either partially or fully reverted. For example, the *gcn4*Δ/Δ mutant had attenuated virulence in *G. mellonella* and the reintroduction of *GCN4* restored the phenotype (Figure 10). In mice, the *gcn4*Δ/Δ mutant exhibited attenuated virulence and the revertant strain reverted to virulence levels close to the wild type (Figure 10C). The partial recovery of wild type phenotype could be explained by the fact that only one copy of the gene was present in the revertant strain, as opposed to two copies in the wild type. In fungal burden assays, the mutant showed lower fungal burden levels in both animal models and the revertant strain showed levels similar to the wild type. This demonstrates that the results obtained here are indeed due only to the deletion of *GCN4*, and support the observation that *GCN4* is necessary for virulence in the two infection models used.

## Discussion

*MBF1* was previously shown to be necessary for virulence of *C. albicans* in two models of systemic infection (Amorim-Vaz et al., 2015a), but its role was not yet understood. Here, we demonstrated its implication in resistance to amino acid starvation. RNA-sequencing of the *mbf1*Δ/Δ mutant was carried out in order to elucidate which genes are controlled by this putative transcriptional coactivator. *In vitro*, during logarithmic growth in rich medium, *the mbf1*Δ/Δ mutant expressed lower levels of genes involved in transcriptional regulation, response to nutritional stress, to host defenses, in adhesion and in biofilm formation, according to a GO term enrichment analysis. These processes were shown by several studies to be upregulated in *C. albicans* during infection of mice kidneys (Amorim-Vaz et al., 2015b). A defect in activating these processes could explain or at least contribute to the reduced virulence of the *mbf1*Δ/Δ mutant during systemic infection of mice and *G. mellonella* (Amorim-Vaz et al., 2015b). In addition, a GSEA showed that gene regulation in the *mbf1*Δ/Δ mutant is inversely correlated with gene regulation in the wild type in several conditions mimicking stresses encountered *in vivo* (such as hypoxia or interaction with immune cells). Nevertheless, it has been shown in the past that regulatory networks of gene expression can be alternatively wired in different conditions, such as those prevailing *in vitro* and *in vivo* (Kumamoto, 2008; Fanning et al., 2012). Therefore, we analyzed gene regulation of the *mbf1*Δ/Δ mutant *in vivo* to verify whether the observations made *in vitro* were also valid during infection. *In vivo* transcriptional analysis to the *mbf1*Δ/Δ mutant was challenging given the low levels of fungal burdens in host organs, making it difficult to obtain sufficient RNA for sequencing. The SureSelect enrichment method made this analysis possible, but it also revealed limitations. When dealing with mutant strains producing low fungal burden levels in animals, the capture of *C. albicans*-specific RNAs is less efficient than for wild type strains. Accordingly, the analysis of *MBF1* transcriptome *in vivo* was more difficult by high variability of results. We also only selected kidneys as target organs of *C. albicans* infections since it is well-known that these organs usually contain the highest fungal loads (Amorim-Vaz et al., 2015b). Even with these limitations, some observations could be made. In this case, the gene regulation observed in the *mbf1*Δ/Δ mutant *in vivo* relative to *in vitro* was compared to the gene regulation observed in the wild type *in vivo* relative to *in vitro*. In other words, we looked at how the two strains differ in their adaptation to *in vivo* conditions. While in the wild type upregulation of genes involved in fatty acid metabolism, carbohydrate transport and the glyoxylate cycle can be observed, the *mbf1*Δ/Δ mutant failed to activate these processes to the same extent. *Candida albicans* typically switches its carbon sources to two-carbon compounds or fatty acids *in vivo*, decreasing the utilization of sugars like glucose which are scarce in that environment (Lorenz and Fink, 2002; Amorim-Vaz et al., 2015b). Here, we observed that the *mbf1*Δ/Δ mutant was unable (or less able) to make this switch on the transcriptional level. In a previous study, no phenotype was observed *in vitro* for the *mbf1*Δ/Δ mutant grown in alternative carbon sources (Amorim-Vaz et al., 2015a). These tests were carried out with cells grown in rich medium (YEPD) and then spotted on media containing different carbon sources. Here we found a phenotype for the *mbf1*Δ/Δ mutant in amino acid starvation, but only after growing cells in medium lacking histidine (depleting the cell's reserves) and then spotting on medium without histidine and containing 3-AT. As an alternative, this could indicate that *MBF1* is involved in these processes only *in vivo*. Genes related to ribosome biogenesis and RNA processing were upregulated in the *mbf1*Δ/Δ mutant in comparison to the wild type, possibly indicating a failure in decreasing protein synthesis in an environment poor in available amino acids.

Taken together, the observations discussed above suggest a role of *MBF1* in the adaptation of the metabolism to the environment. Supporting this hypothesis is the observation that the *mbf1*Δ/Δ mutant was more susceptible than the wild type in the presence of 3-AT. If Mbf1 is involved in regulation of protein synthesis, it is likely to interact at some level with a master regulator of protein synthesis and response to starvation in *C. albicans* such as Gcn4. Therefore, we investigated whether gene regulation in the *mbf1*Δ/Δ mutant during amino acid starvation reflected the same gene regulation patterns observed in a *gcn4*Δ/Δ mutant. A significant positive correlation was observed between the transcriptomic changes of the two mutants (Figure 5). The transcriptional regulation observed in the *gcn4*Δ/Δ mutant, namely the failure to downregulate RNA processing and to upregulate amino acid biosynthesis during amino acid starvation, is in agreement with previous knowledge on this transcription factor in *C. albicans* (Tripathi et al., 2002) and in *S. cerevisiae* (Natarajan et al., 2001). The genes involved in these processes were generally regulated in the *mbf1*Δ/Δ mutant in the same direction (up- or down-regulation) as in the *gcn4*Δ/Δ mutant, although not as strongly and therefore did not pass the imposed threshold. This could indicate that *MBF1* participates in the regulation of these processes. However, since Mbf1 may be one among several transcriptional co-activators of *GCN4*, its contribution in these processes may be only partial.

To further elucidate the possible interaction between *MBF1* and *GCN4*, we tested the impact of *GCN4* in virulence and found that it was necessary for virulence in two models of systemic infection. *GCN4* is a master regulator of response to starvation. Upon limited amino acid availability from the external environment, *GCN4* slows down protein synthesis, activates amino acid biosynthesis and promotes nucleophagy in order to recycle cellular pools of nitrogen (reviewed in Philpott et al., 2012). The environment of infection is predicted to be a harsh environment, with limited nutrient availability intensified by an immune system that resorts to nutritional immunity (Potrykus et al., 2014). It was therefore surprising that a previous work indicated that *GCN4* was not necessary for virulence (Brand et al., 2004). Here, we showed strong evidence supporting a role of *GCN4* in virulence, and propose that the results in Brand et al. (2004) were compromised by unexpected effects of the strain construction methods used. These findings demonstrate the importance of control of amino acid biosynthesis for *C. albicans* virulence, which in turn reveal that limitation of amino acid availability is an important stress that *C. albicans* has to face and overcome in the systemic environment. Supporting this observation, *in vivo* transcriptomics showed regulation of several genes from amino acid biosynthetic pathways, both in our work and in the work of Xu et al. (2015) (reviewed in Fanning and Mitchell, 2017).

Mbf1 is conserved in eukaryotes and has been observed in the nucleus in other organisms (Kabe et al., 1999; Mariotti et al., 2000; Busk et al., 2003; Zanetti et al., 2003; Ballabio et al., 2004; Jindra et al., 2004; Sugikawa et al., 2005). Mammalian Mbf1 was shown to need phosphorylation in order to be translocated to the nucleus (Mariotti et al., 2000; Ballabio et al., 2004), which occurred following a stress response (Busk et al., 2003). We did not observe an organelle preference of Mbf1 in the present work. These data are however consistent with the idea that Mbf1 may interact with proteins localized in multiple cell compartments. We showed at least here that Mbf1 could interact at least with Gcn4 using an indirect method based on the reconstitution of fluorescent fusion proteins. Probing the systematic protein interactome with Mbf1 should be undertaken in the future to complement our analysis.

In conclusion, this work provided further insights into the understanding of *MBF1* functions in *C. albicans*. *Candida albicans* is a common commensal of human mucosae, but also has the ability to infect the bloodstream and internal organs, where it faces conditions and stresses that greatly differ from those encountered in the mucosae. For this transition from commensalism to pathogenicity to be successful, this microorganism needs to accurately control gene expression in order to respond to the challenges of these different environments. Through the analysis of *MBF1*, our work contributed to understand the strategies employed by *C. albicans* for an appropriate adaptation to the environment of systemic infections.

## Data Availability Statement

The datasets presented in this study can be found in online repositories. The names of the repository/repositories and accession number(s) can be found at: https://www.ncbi.nlm.nih.gov/, PRJNA350368.

## Ethics Statement

The animal study was reviewed and approved by Affaires Vétérinaires du Canton de Vaud, Switzerland, authorization n° 1734.3.

## Author Contributions

SA-V performed experiments. VT and DS performed data analysis. AC, MP, and DS designed experiments. All authors contributed to the article and approved the submitted version.

## Conflict of Interest

The authors declare that the research was conducted in the absence of any commercial or financial relationships that could be construed as a potential conflict of interest.
