# Supplementary material for: Function Analysis of MBF1, a Factor Involved in the Response to Amino Acid Starvation and Virulence in Candida albicans
Source: Front Fungal Biol. 2021 Mar 15;2:658899. doi: 10.3389/ffunb.2021.658899 (PMC10512259; doi:10.3389/ffunb.2021.658899)
Supplement: Supplementary file 6 [file Data_Sheet_6.docx]

Table S1: List of primers

| Usage | Name | Sequence |
| --- | --- | --- |
|  | orf19.3294-5F-Apa | gcgaaagggcccCGTGATTTGACTTCAATGTCA |
|  | orf19.3294-3R-Xho | CGCAAACTCGAGATACCGCTGAAAAATGTTGAC |
|  | orf19.3294-3F-SacII | cgcaaaccgcggGGGTTTCAATGATTGACGAAT |
|  | orf19.3294-3R-SacI | cgcaaagagctcATACCGCTGAAAAATGTTGAC |
| Primers used for | GCN4-kpn | ATCTGGTACCAACTTATCATAAATTATTATTAT |
| strain construction | GCN4-XhoI | CAAAGCTCGAGAATGTAATGTAATTTAATTTA |
|  | GCN4-SacII | AAGAGTCCGCGGTATTCAATTTTAAAAAGTTAC |
|  | GCN4-SacI | CCTCTTTTGAGCTCACATATGTTGTATACACCAATGA |
|  | GCN4-XhoIrev2 | ACTATCCTCGAGTCAGTTCACATATGTTGTATACA |
|  | MBF1_atg_SalI | CCAAGTCGACATGTCTTCAGATTGGG |
|  | MBF1_nostop_sph | CCAAGCATGCATTTTTTCTTAGCAAAC |
|  | MBF1_BiFC_NF | AGCCTAGGGACCCGGATGTCTTCAGATTGGGATTCAGT |
|  | MBF1_BiFC_NR | ACGCGTTTAGGCGCGTTATTTTTTCTTAGCAAACAAAGGT |
|  | BiFC-GCN4-NF | AGCCTAGGGACCCGGATGCCTGCTACTACTCCT |
|  | BiFC-GCN4-NR | ACGCGTTTAGGCGCGTTAAAATTGAATACCATTAACTCTT |
|  | 3294-60 | AAATGACACCGTTGAC |
| Sequencing primers | GCN4_seq_+350F | GTTGCTATAGCTGGTGCTAC |
|  | GCN4_seq_+800R | GCTCTGGATCTTCTAGCAGC |
|  | ACT1-RT-F | ATAACGGTTCTGGTATGT |
|  | ACT1-RT-R | CCTTGATGTCTTGGTCTA |
|  | ACT1-RT-P | CGGTGACGACGCTCCAAG |
|  | BIO2-RT-F | GGACATTGCTAGAGATGCGA |
|  | BIO2-RT-R | AGGCAGACTTTCTTCCTTGC |
|  | BIO2-RT-P | TCCAAGCGGAACCAAGGCAA |
|  | HWP1-F | TCAGTTCCACTCATGCAACCA |
|  | HWP1-R | ATAGCAGCACCGAAAGTCAATC |
| Primers and probes | HWP1-P | TGCCAATTACTCAAGTGTCGCTCCTATTTC |
| used for qPCR | RBT1-RT-F | TTTCGAAAGCAACTGTCCTG |
|  | RBT1-RT-R | GGCTTTGACTTGAGCATTGA |
|  | RBT1-RT-P | CAATGCCGTTTCTAACGCAATTGG |
|  | STP4-RT-F | GTTATCAATGGCCGTAACCC |
|  | STP4-RT-R | TGTGGTGGAGAAGTCATGGT |
|  | STP4-RT-P | CGCCATCTCCAACATCTACGCC |
|  | YWP1-F | CAGGTACTTTGACACCGGAAAAT |
|  | YWP1-R | GAATCAAAGCTAAAGCCAAGACTGA |
|  | YWP1-P | TGCCGCCGCCGCTTCA |
|  | ZRT1-F | TCAACTTCCTTGAATGCTTCTACAA |
|  | ZRT1-R | TGGACCGAATTATGAAAGTGACA |
|  | ZRT1-P | CCACACCACCACTGAACCTGTCTGC |

Table S2: List of plasmids

| Plasmid | Origin | Reference | Description |
| --- | --- | --- | --- |
| pSV5 | pAC286 | (Amorim-Vaz et al., 2015) | Insertion of the ApaI-XhoI fragment amplified from SC5314 with primers orf19.3294-5F-Apa and orf19.3294-3R-Xho (*MBF1* reversion cassette) |
| pDS1907 | pSFS2A | (Reuss et al., 2004) | Insertion of the KpnI-XhoI fragment amplified from SC5314 with primers GCN4-kpn and GCN4-XhoI (5’-UTR of *GCN4*) |
| pDS1911 | pDS1907 | This study | Insertion of the SacI-SacII fragment amplified from SC5314 with primers GCN4-SacII and GCN4-SacI (3’-UTR of *GCN4*) (*GCN4* deletion cassette) |
| pDS1917 | pDS1911 | This study | Insertion of the KpnI-XhoI fragment amplified from SC5314 with primers GCN4-kpn and GCN4-XhoIrev2 (*GCN4* reversion cassette) |
| pSV17 | pDS1202 | (Coste et al., 2004) | Insertion of the SalI-SphI fragment amplified with primers MBF1_atg_SalI and MBF1_nostop_sph |
| pDS2121 | BiFC3 | (Subotić et al., 2017) | BiFC3-derived containing N-terminal tagging of *MBF1* with the C-terminal end of yEmVenus, CV-MBF1 |
| pDS2126 | BiFC1 | (Subotić et al., 2017) | BiFC1-derived containing N-terminal tagging of *GCN4* with the N-terminal end of yEmVenus, NV-MBF1 |

Table S3: List of strains

| Strain | Parent | Genotype | Reference |
| --- | --- | --- | --- |
| SC5314 | - | Wild type | (Gillum et al., 1984) |
| ACY367 | ACY365 | *mbf1*Δ::*FRT*/*mbf1*Δ::*FRT* | (Amorim-Vaz et al., 2015) |
| SVY7 | ACY367 | *mbf1*Δ::*FRT*/*MBF1*::*FRT-SAT1-FRT* | This study |
| SVY24 | SVY7 | *mbf1*Δ::*FRT*/*MBF1::FRT* | This study |
| DSY4836 | SC5314 | *gcn4*Δ::*FRT-SAT1-FRT*/*GCN4* | This study |
| DSY4838 | DSY4836 | *gcn4*Δ::*FRT*/*GCN4* | This study |
| DSY4842 | DSY4838 | *gcn4*Δ::*FRT-SAT1-FRT*/*gcn4*Δ::*FRT* | This study |
| DSY4843 | DSY4842 | *gcn4*Δ::*FRT*/*gcn4*Δ::*FRT* | This study |
| DSY4924 | DSY4843 | *gcn4*Δ::*FRT*/*GCN4*::*FRT-SAT1-FRT* | This study |
| DSY4925 | DSY4924 | *gcn4*Δ::*FRT*/*GCN4*::*FRT* | This study |
| DSY4837 | ACY367 | *mbf1*Δ::*FRT*/*mbf1*Δ::*FRT*, *gcn4*Δ::*FRT-SAT1-FRT*/*GCN4* | This study |
| DSY4839 | DSY4837 | *mbf1*Δ::*FRT*/*mbf1*Δ::*FRT*, *gcn4*Δ::*FRT*/*GCN4* | This study |
| DSY4841 | DSY4839 | *mbf1*Δ::*FRT*/*mbf1*Δ::*FRT*, *gcn4*Δ::*FRT-SAT1-FRT*/*gcn4*Δ::*FRT* | This study |
| DSY4917 | DSY4841 | *mbf1*Δ::*FRT*/*mbf1*Δ::*FRT*, *gcn4*Δ::*FRT*/*gcn4*Δ::*FRT* | This study |
| DSY4920 | DSY4917 | *mbf1*Δ::*FRT*/*mbf1*Δ::*FRT*, *gcn4*Δ::*FRT*/*GCN4::FRT-SAT1-FRT* | This study |
| DSY4922 | DSY4920 | *mbf1*Δ::*FRT*/*mbf1*Δ::*FRT*, *gcn4*Δ::*FRT*/*GCN4::FRT* | This study |
| DSY4926 | DSY4922 | *mbf1*Δ::*FRT*/*MBF1*::*FRT-SAT1-FRT*, *gcn4*Δ::*FRT*/*GCN4::FRT* | This study |
| ACY392 | ACY361 | *mbf1*Δ::*FRT*/*MBF1, ura3*Δ::*FRT-SAT1-FRT*/*URA3* | This study |
| ACY393 | ACY392 | *mbf1*Δ::*FRT*/*MBF1, ura3*Δ::*FRT*/*URA3* | This study |
| ACY394 | ACY393 | *mbf1*Δ::*FRT*/*MBF1, ura3*Δ::*FRT-SAT1-FRT*/*ura3*Δ::*FRT* | This study |
| ACY395 | ACY394 | *mbf1*Δ::*FRT*/*MBF1, ura3*Δ::*FRT*/*ura3*Δ::*FRT* | This study |
| SVY25 | ACY395 | *mbf1*Δ::*FRT*/*mbf1*Δ::*FRT-SAT1-FRT, ura3*Δ::*FRT*/*ura3*Δ::*FRT* | This study |
| SVY26 | SVY25 | *mbf1*Δ::*FRT*/*mbf1*Δ::*FRT, ura3*Δ::*FRT*/*ura3*Δ::*FRT,*  *RPS10/rps10*::*MBF1-GFP*::*URA3* | This study |
| SN152 | SC5314 | *arg4*∆*/arg4*∆ *leu2*∆*/leu2*∆ *his1*∆*/his1*∆ *URA3/ura3*∆*:: imm434 IRO1/ iro1::imm434* | (Noble and Johnson, 2005) |
| DSY5529 | SN152 | pDS2121::*ARG4* | This study |
| DSY5544 | DSY5529 | pDS2121::*ARG4*  pDS2126::*LEU2* | This study |
| DSY5552 | SN152 | BiFC3::*ARG4* | This study |
| DSY5551 | DSY5552 | pDS2126::*LEU2* BiFC3::*ARG4* | This study |
| DSY5554 | DSY5529 | pDS2121::*ARG4* BiFC1::*LEU2* | This study |

Table S4: Volume of data obtained after RNA-seq of *in vitro* and *in vivo* samples of wild type (WT) and *mbf1*∆/∆ mutant (average values for each condition)

|  | WT *in vitro* | WT 16 h p.i. | WT 48 h p.i. | *mbf1*∆/∆ *in vitro* | *mbf1*∆/∆ 16 h p.i. | *mbf1*∆/∆ 48 h p.i. |
| --- | --- | --- | --- | --- | --- | --- |
| Reads aligned to *C. albicans* (millions) | 35.5 | 15.9 | 35.1 | 30.8 | 4.5 | 27.1 |
| % of total reads aligned to *C. albicans* | 85.9 | 45.7 | 83.4 | 87.6 | 17.4 | 64.9 |

**References**

Amorim-Vaz, S., Delarze, E., Ischer, F., Sanglard, D., and Coste, A.T. (2015). Examining the virulence of *Candida albicans* transcription factor mutants using *Galleria mellonella* and mouse infection models. *Front Microbiol* 6. doi: ARTN 367

DOI 10.3389/fmicb.2015.00367.

Coste, A.T., Karababa, M., Ischer, F., Bille, J., and Sanglard, D. (2004). TAC1, transcriptional activator of CDR genes, is a new transcription factor involved in the regulation of Candida albicans ABC transporters CDR1 and CDR2. *Eukaryot Cell* 3(6)**,** 1639-1652. doi: 10.1128/EC.3.6.1639-1652.2004.

Gillum, A.M., Tsay, E.Y., and Kirsch, D.R. (1984). Isolation of the Candida albicans gene for orotidine-5'-phosphate decarboxylase by complementation of S. cerevisiae ura3 and E. coli pyrF mutations. *Mol Gen Genet* 198(1)**,** 179-182.

Noble, S.M., and Johnson, A.D. (2005). Strains and strategies for large-scale gene deletion studies of the diploid human fungal pathogen Candida albicans. *Eukaryot Cell* 4(2)**,** 298-309. doi: 10.1128/EC.4.2.298-309.2005.

Reuss, O., Vik, A., Kolter, R., and Morschhauser, J. (2004). The SAT1 flipper, an optimized tool for gene disruption in Candida albicans. *Gene* 341**,** 119-127.

Subotić, A., Swinnen, E., Demuyser, L., De Keersmaecker, H., Mizuno, H., Tournu, H., et al. (2017). A Bimolecular Fluorescence Complementation Tool for Identification of Protein-Protein Interactions in Candida albicans. *G3 (Bethesda, Md.)* 7(10)**,** 3509-3520. doi: 10.1534/g3.117.300149.
