# Supplementary material for: Function Analysis of MBF1, a Factor Involved in the Response to Amino Acid Starvation and Virulence in Candida albicans
Source: Front Fungal Biol. 2021 Mar 15;2:658899. doi: 10.3389/ffunb.2021.658899 (PMC10512259; doi:10.3389/ffunb.2021.658899)
Supplement: Supplementary file 7 [file Data_Sheet_7.docx]

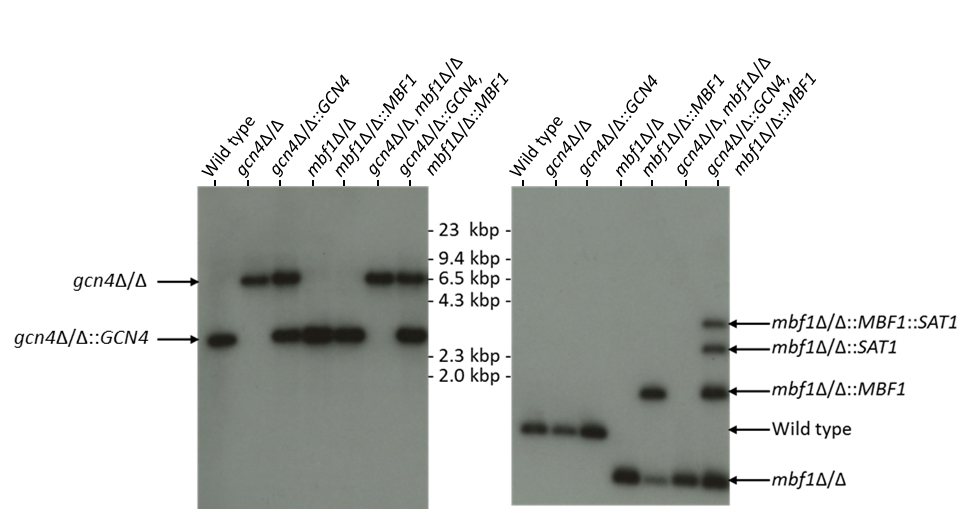


Figure S1: Southern blot with strains used in this study. The fragments detected correspond to the expected sizes calculated in restriction maps. Panel A: a probe for *GCN4* 5’-region was used following HindIII DNA digestion. This probe was amplified by PCR from SC5314 with primers GCN4-knp and GCN4-Xho (Table S1). Panel B: a probe for *MBF1* 3’-region was used following BglII DNA digestion. This probe was amplified by PCR from SC5314 with primers orf19.3294-3F-SacII and orf19.3294-3R-SacI (Table S1). Wild type: SC5314; *gcn4*∆/∆: DSY4843; *gcn4*∆/∆::*GCN4*: DSY4925; *mbf1*∆/∆: ACY367; *mbf1*∆/∆::*MBF1*: SVY24; *gcn4*∆/∆, *mbf1*∆/∆: DSY4917; *gcn4*∆/∆::*GCN4*, *mbf1*∆/∆::*MBF1*: DSY4926.


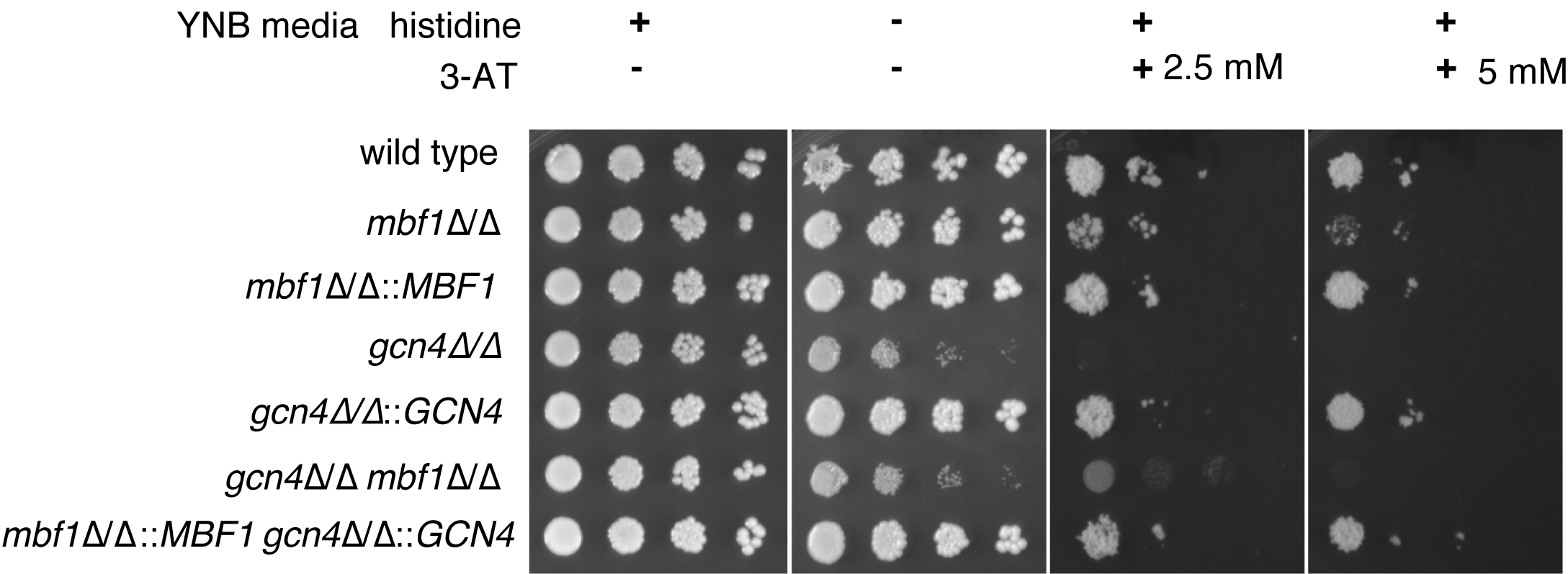


**Figure S2:** Assessment of the influence of *MBF1* on *C. albicans* resistance to amino acid starvation on solid media. Strains were grown in liquid media lacking histidine before serial 10-fold dilutions of each strain were spotted on YNB + CSM, YNB -his is or YNB -his + 3-AT (2.5 and 5 mM). Plates were incubated at 35°C and pictures taken 48 hours later. Strain designations: Wild type – SC5314; *mbf1*∆/∆ - ACY367; *mbf1*∆/∆::*MBF1* – SVY24; *gcn4*∆/∆ - DSY4843; *gcn4*∆/∆::*GCN4* – DSY4925; *gcn4*∆/∆, *mbf1*∆/∆ – DSY4917; *gcn4*∆/∆::*GCN4*, *mbf1*∆/∆::*MBF1* – DSY4926.


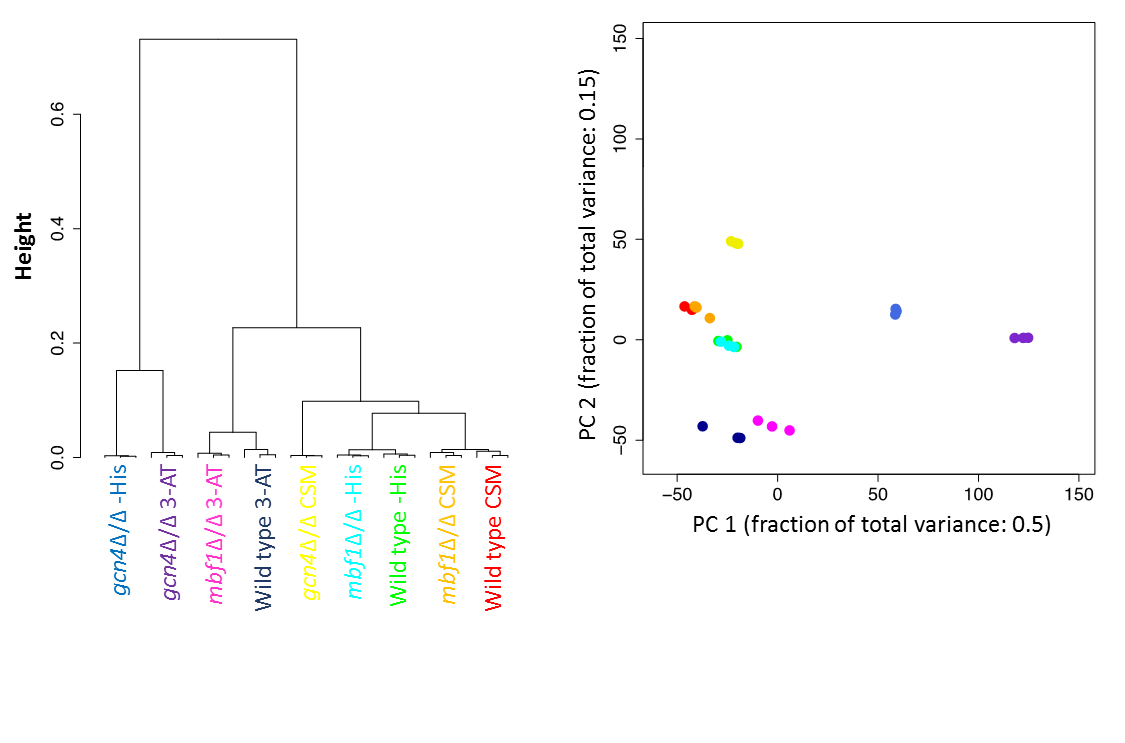


Figure S3: Hierarchical clustering (left) and PCA (right) of samples used to characterize the influence of *MBF1* and *GCN4* in transcription during amino acid limitation. The response of wild type (SC5314), *mbf1*∆/∆ mutant (ACY367) and *gcn4*∆/∆ mutant (DSY4843) were compared during growth in YNB medium supplemented with all amino acids (CSM), YNB medium supplemented with all amino acids except histidine (-his) or YNB medium supplemented with all amino acids except histidine and supplemented with 1 mM 3-AT (3-AT). Clustering and PCA were performed in R (version 3.2.2.) using Voom-transformed and normalized gene counts. The 5548 *C. albicans* genes with at least 1 count per million in at least one sample were used for clustering and PCA.

Raw data for comparison of fold-change obtained by RNA-seq and by qPCR for six genes

|  |  | ***BIO2*** | ***HWP1*** | ***RBT1*** | ***STP4*** | ***YWP1*** | ***ZRT1*** |
| --- | --- | --- | --- | --- | --- | --- | --- |
| **FC WT 3-AT/CSM** | **qPCR** ^a^ | 2.87 | 12.47 | 17.74 | 0.15 | 0.15 | 3.96 |
|  | **RNA-seq** | 4.06 | 9.86 | 10.69 | 0.19 | 0.31 | 5.85 |
| **FC *mbf1* 3-AT/CSM** | **qPCR** | 2.08 | 6.88 | 8.27 | 0.63 | 0.10 | 4.15 |
|  | **RNA-seq** | 1.52 | 1.55 | 5.28 | 0.71 | 0.18 | 2.97 |
| **FC *gcn4* 3-AT/CSM** | **qPCR** | 0.41 | 3.97 | 0.46 | 0.10 | 0.23 | 8.61 |
|  | **RNA-seq** | 0.08 | 3.70 | 0.56 | 0.06 | 0.23 | 7.11 |

^a^ Fold change for qPCR results was calculated as 2^-ΔΔCT^.

Figure S4: Correlation between fold-change obtained by RNA-seq and by qPCR. Fold-change for qPCR results was calculated as 2^-ΔΔCT^. Values were plotted and Pearson correlation was calculated using GraphPad Prism software (version 8.4.3). r^2^ = 0.86, p ≤ 0.0001. Raw data are given in the included Table.


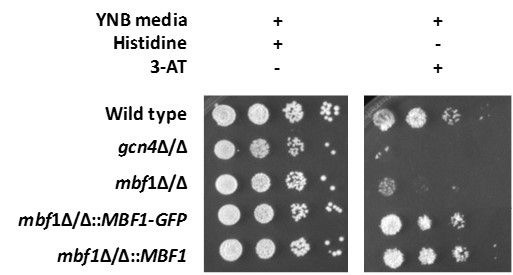


Figure S5: Verification of Mbf1-GFP function. Strains were grown in liquid media lacking histidine before serial 10-fold dilutions of each strain were spotted on YNB + CSM or YNB –his + 3-AT (2.5 mM). Plates were incubated at 35°C and pictures taken after 72 hours incubation. Representative results are shown.
